# Never-germinating *Arabidopsis* seeds with LbCas12a-induced mutations in 6 clade A type 2C protein phosphatase genes

**DOI:** 10.1093/plphys/kiaf315

**Published:** 2025-07-17

**Authors:** Cuiping Xin, Yu Lu, Syeda Leeda Gul, Wei Sun, Zhenghong Cao, Xiangchao Kong, Kexin Fan, Siyun Li, Xiaohan Liu, Xue-Chen Wang, Qi-Jun Chen

**Affiliations:** State Key Laboratory of Plant Environmental Resilience, College of Biological Sciences, China Agricultural University, Beijing 100193, China; State Key Laboratory of Plant Environmental Resilience, College of Biological Sciences, China Agricultural University, Beijing 100193, China; State Key Laboratory of Plant Environmental Resilience, College of Biological Sciences, China Agricultural University, Beijing 100193, China; State Key Laboratory of Plant Environmental Resilience, College of Biological Sciences, China Agricultural University, Beijing 100193, China; State Key Laboratory of Plant Environmental Resilience, College of Biological Sciences, China Agricultural University, Beijing 100193, China; State Key Laboratory of Plant Environmental Resilience, College of Biological Sciences, China Agricultural University, Beijing 100193, China; State Key Laboratory of Plant Environmental Resilience, College of Biological Sciences, China Agricultural University, Beijing 100193, China; State Key Laboratory of Plant Environmental Resilience, College of Biological Sciences, China Agricultural University, Beijing 100193, China; State Key Laboratory of Plant Environmental Resilience, College of Biological Sciences, China Agricultural University, Beijing 100193, China; State Key Laboratory of Plant Environmental Resilience, College of Biological Sciences, China Agricultural University, Beijing 100193, China; State Key Laboratory of Plant Environmental Resilience, College of Biological Sciences, China Agricultural University, Beijing 100193, China; Center for Crop Functional Genomics and Molecular Breeding, China Agricultural University, Beijing 100193, China

## Abstract

Cas12 nucleases, such as Cas12a, Cas12i, and Cas12f, are genome-editing tools that possess several unique attributes. However, the potential of various Cas12 variants for multiplex genome editing in *Arabidopsis* (*Arabidopsis thaliana*) remains insufficiently characterized. In this report, we systematically evaluated 18 additional targets and demonstrated that the LbCas12a variant carrying D156R and E795L mutations exhibits minimal target bias. Furthermore, we achieved an editing efficiency of at least 73.8% (45/61) in generating T1 homozygous sextuple mutants, with more than half of these mutants exhibiting a complete seed germination arrest phenotype. Comparative analysis of 7 LbCas12a variants revealed that the optimization of nuclear localization sequences, rather than codon usage, is fundamental for improved editing efficiency, and that the E795L mutation had synergistic effects with other mutations in highly efficient LbCas12a variants. Further investigation into 1 Cas12i3 and 2 AsCas12f variants showed that the Cas12i3 variant also exhibits sufficiently high editing efficiency in *Arabidopsis*, although additional refinements were required to mitigate its target bias. Collectively, in this study, we developed the most efficient clustered regularly interspaced short palindromic repeat (CRISPR)/CRISPR-associated nuclease (Cas) tool for multiplex genome editing in *Arabidopsis*, as demonstrated by the highly efficient generation of never-germinating seeds harboring mutations in 6 clade A type 2C protein phosphatase genes.

## Teaser

An optimized LbCas12a variant efficiently generates seemingly never-germinating *Arabidopsis* seeds carrying homozygous or biallelic mutations in 6 of 9 clade A type 2C protein phosphatase genes.

## Introduction

Cas12a (Cpf1), an endonuclease belonging to the Class 2 Type V-A clustered regularly interspaced short palindromic repeat (CRISPR)/CRISPR-associated nuclease (Cas) system, has garnered considerable attention as a genome-editing tool due to its distinct molecular properties (Zetsche et al. 2015, 2017; Makarova et al. 2020). Although LbCas12a has been employed for genome editing in plants (Tang et al. 2017; Wang et al. 2017, 2021; Xu et al. 2017, 2019; Li et al. 2018, 2020; Zhong et al. 2018; Bernabe-Orts et al. 2019; Lee et al. 2019; Malzahn et al. 2019; Wolter and Puchta 2019; Merker et al. 2020; Schindele and Puchta 2020; Zhang et al. 2022; Schindele et al. 2023; Su et al. 2023; Zhou et al. 2023), its practical application remains constrained by intrinsic limitations, including temperature sensitivity and suboptimal cleavage activity. These factors substantially impede its efficiency, necessitating further optimization. Fortunately, extensive efforts have sought to overcome these restrictions (Lee et al. 2019; Liu et al. 2019a; Zhang et al. 2021, 2023; Guo et al. 2022; Huang et al. 2022; Ma et al. 2022).

In our previous report, we demonstrated that the LbCas12a variant, ttLbCas12a Ultra V2 (ttLbUV2), harboring the D156R and E795L mutations derived from the low-temperature-tolerant and highly active variants, respectively, along with optimized nuclear localization signal (NLS) sequences and codon usage, greatly enhanced the editing efficiency in *Arabidopsis* (Xin et al. 2024). However, evidence is lacking for the capability of ttLbUV2 in multiplex genome editing. Moreover, it remains unclear which of the 2 optimized strategies, varying NLS sequences or optimizing codon usage, is the primary factor responsible for the enhanced editing efficiency, and it is also uncertain which of the reported highly active variants, including ttLbUV2, is the most efficient for multiplex genome editing.

Compared to LbCas12a, Cas12i3, a Class 2 Type V-I CRISPR/Cas system, features a smaller protein size (1045-aa vs. 1228-aa) and a more flexible PAM preference (TTN vs. TTTV) (Bai et al. 2024; Lv et al. 2024), which represents advantages in size-sensitive delivery of reagents for genome editing, such as virus-mediated delivery, and PAM-sensitive applications, such as base editing and prime editing (Liang et al. 2024; Lv et al. 2024). AsCas12f1 (422 aa), one of the smallest Cas nucleases, is a Class 2 Type V-F Cas nuclease and recognizes YTTN or NTTR PAMs (Hino et al. 2023). While a Cas12i3 and 2 Cas12f variants were reported to induce efficient genome editing in plants (Duan et al. 2024; Wang et al. 2024, 2025; Ye et al. 2024), evaluating these variants in *Arabidopsis* could facilitate the development of more efficient tools for plant genome editing and provide clues for their further optimization if necessary.

In this report, we systematically assessed the target bias of the ttLbUV2 variant for genome editing by testing 18 additional targets, presented its capability to generate homozygous sextuple mutants in 9 clade A PP2Cs, determined the primary factor responsible for its enhanced activity between 2 optimization strategies, and identified the most efficient one of the 7 LbCas12a variants. Additionally, we also tested an optimized Cas12i3 and 2 AsCas12f variants to explore their genome-editing potential in *Arabidopsis*. Our results demonstrate that the optimized LbCas12a variant exhibits minimal target bias and constitutes the most efficient CRISPR/Cas tool for multiplex genome editing in *Arabidopsis* and efficiently generates never-germinating seeds harboring mutations in 6 clade A PP2Cs.

## Results

### The optimized LbCas12a variant was highly efficient and exhibited low target bias for genome editing in *Arabidopsis*

**Figure 1.**
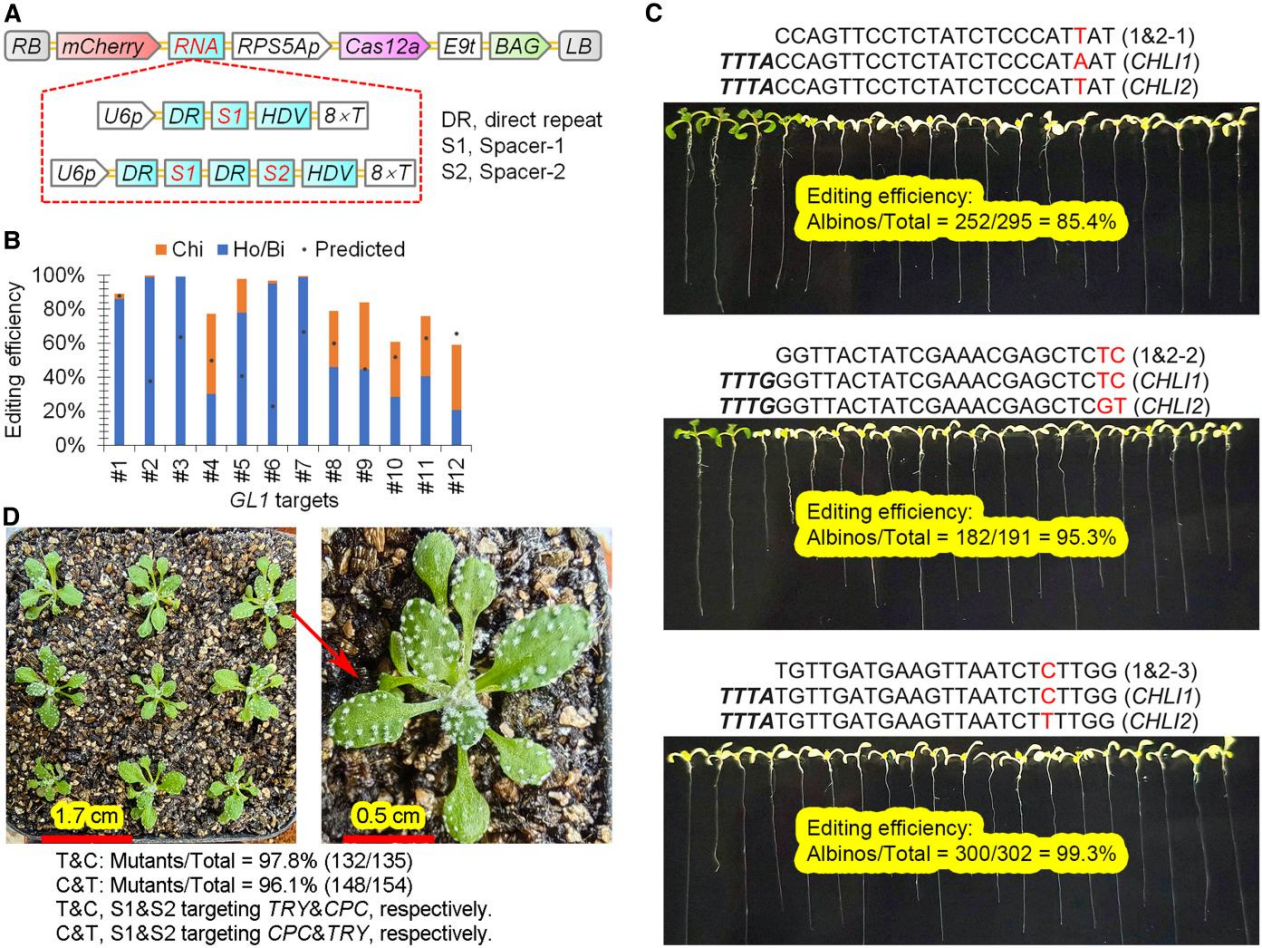
Editing efficiency of the optimized LbCas12a variant across 18 single or dual targets. **A)** T-DNA structures of binary vectors. RB and LB, right and left T-DNA borders, respectively. *RPS5Ap*, *Arabidopsis RPS5A* promoter; *rbcS-E9t*, pea *rbcS E9* terminator; *BAG*, *Bar-F2A-GAT*, Basta and glyphosate-resistant marker gene; *U6p*, *Arabidopsis U6-26* promoter; *tGly*, *tRNA(Gly)*; *HDV*, hepatitis delta virus ribozyme. **B)** Editing efficiency of ttLbUV2 across 12 GL1 targets from 2,238 T1 transgenic lines. Ho/Bi, phenotypically homozygous or biallelic mutants exhibiting complete glabrous phenotypes. Chi, phenotypically chimeric mutants with partial glabrous phenotypes. Predicted scores indicate computationally estimated editing efficiency. Data for targets #1 (GL1-1) and #2 (GL1-2) were previously reported, where GL1-1 was edited using the less efficient ttLbCas12a Ultra variant in combination with the U6-tRNA(Gly) cassette (Xin et al. 2024). **C)** Efficiency of generating homozygous or biallelic double mutants in *CHLI1* and *CHLI2*. Alignment of crRNA sequences with their corresponding dual targets is indicated, with mismatched nucleotides highlighted in red. The PAMs in targets are indicated with bold and italic font. **D)** Efficiency of generating homozygous or biallelic double mutants in *TRY* and *CPC*. The same 2 crRNAs were used in both constructs, with their positions swapped within the tandem arrays.

To further evaluate the editing efficiency and target bias degree of ttLbUV2, an optimized LbCas12a variant described in our previous study (Xin et al. 2024), we analyzed its performance across 10 additional targets in *GL1* (Fig. 1, A and B), 3 pairs of homologous targets for simultaneous mutations in *CHLI1* and *CHLI2* (Fig. 1, A and C), and a pair of targets for simultaneous mutations in *TRY* and *CPC* (Fig. 1, A and D). Phenotypic characterization of these mutants has been well established: *gl1* single mutants exhibit glabrous phenotypes (Xin et al. 2024), *chli1 chli2* double mutants develop albino phenotypes (Wang et al. 2015), and *try cpc* double mutants display clustered trichome phenotypes (Wang et al. 2015). We achieved editing efficiencies that ranged from 20.8% (26/125) to 99.1% (214/216) for homozygous or biallelic mutations across the 10 tested *GL1* targets in T1 transgenic plants (Fig. 1B; Supplementary Table S1). A comparison between experimental and predicted editing efficiencies (Concordet and Haeussler 2018; Kim et al. 2018) across the 12 *GL1* targets (Fig. 1B; Supplementary Table S1) indicated that ttLbUV2 maintained high editing efficiency even at target #6, which had been predicted to have low efficiency. This suggests that ttLbUV2 demonstrated reduced target bias compared to the original system used for efficiency prediction, further supporting its robustness as an effective genome-editing tool.

We employed a strategy for the generation of *chli1 chli2* double mutants by utilizing a single crRNA to simultaneously target both genes. Each of the 3 crRNAs exhibited full complementarity to 1 target gene while containing mismatches at the PAM-distal region of the other (Fig. 1C). Previous studies have established that CRISPR/Cas systems can tolerate mismatches at the PAM-distal site (Zetsche et al. 2015). Using this approach, we achieved high editing efficiencies of 85.4% (252/295), 95.3% (182/191), and 99.3% (300/302) with crRNAs 1&2-1, 1&2-2, and 1&2-3, respectively, for generation of homozygous or biallelic double mutants (Fig. 1C; Supplementary Table S2). These data indicate that 1 or 2 PAM-distal mismatches had no obvious effects on editing efficiency, which is consistent with previous reports and suggests that this strategy can be harnessed for selection of targets for multiplex genome editing.

We adopted a canonical strategy for generating *try cpc* double mutants by utilizing 2 crRNAs to independently target each gene. A single U6 expression cassette was used to drive the expression of the 2 seamlessly fused crRNAs, which were subsequently processed by Cas12a (Wang et al. 2017; Zetsche et al. 2017). To investigate the influence of crRNA positioning within a tandem array, we constructed 2 vectors in which the order of TRY- and CPC-targeting crRNAs was swapped. Both configurations yielded similarly high editing efficiencies, with 97.8% (132/135) for the T&C crRNA array and 96.1% (148/154) for the C&T crRNA array (Fig. 1D; Supplementary Table S3). The results indicate that crRNA positioning within a tandem array does not significantly affect editing efficiency. Together, phenotypic analysis of homozygous or biallelic mutations across all the 18 additional targets in T1 transgenic plants (Fig. 1, B to D; Supplementary Tables S1 to S3) confirms that ttLbUV2 is highly efficient and exhibits negligible target bias for genome editing in *Arabidopsis*.

To validate the heritability of mutations in T1 plants, we isolated T-DNA-free T2 seeds from 12 independent T1 homozygous *try cpc* double-mutant lines, comprising 6 lines derived from pV2-T&C and 6 from pV2-C&T. Phenotypic analysis revealed that all T-DNA-free T2 plants retained the double-mutant phenotype (Supplementary Table S4), demonstrating that mutations in T1 plants are heritable to the subsequent generation. To analyze potential off-target mutagenesis, we computationally identified 3 putative off-target sites that shared 2 or 3 mismatches with the *TRY* target in At2G30432, At2G30420, and At2G30424 (Supplementary Table S5). PCR amplification of genomic fragments spanning these sites was performed on 36 T-DNA-free T2 homozygous double mutants derived from T1 homozygous double mutants carrying pV2-T&C. Sanger sequencing of these amplicons detected no off-target mutations (Supplementary Table S5), supporting the conclusion that careful selection of targets will be able to avoid off-target mutagenesis induced by highly efficient LbCas12a variants.

### The ttLbUV2 variant was highly efficient for the generation of sextuple mutants in clade A PP2Cs in *Arabidopsis*

**Figure 2.**
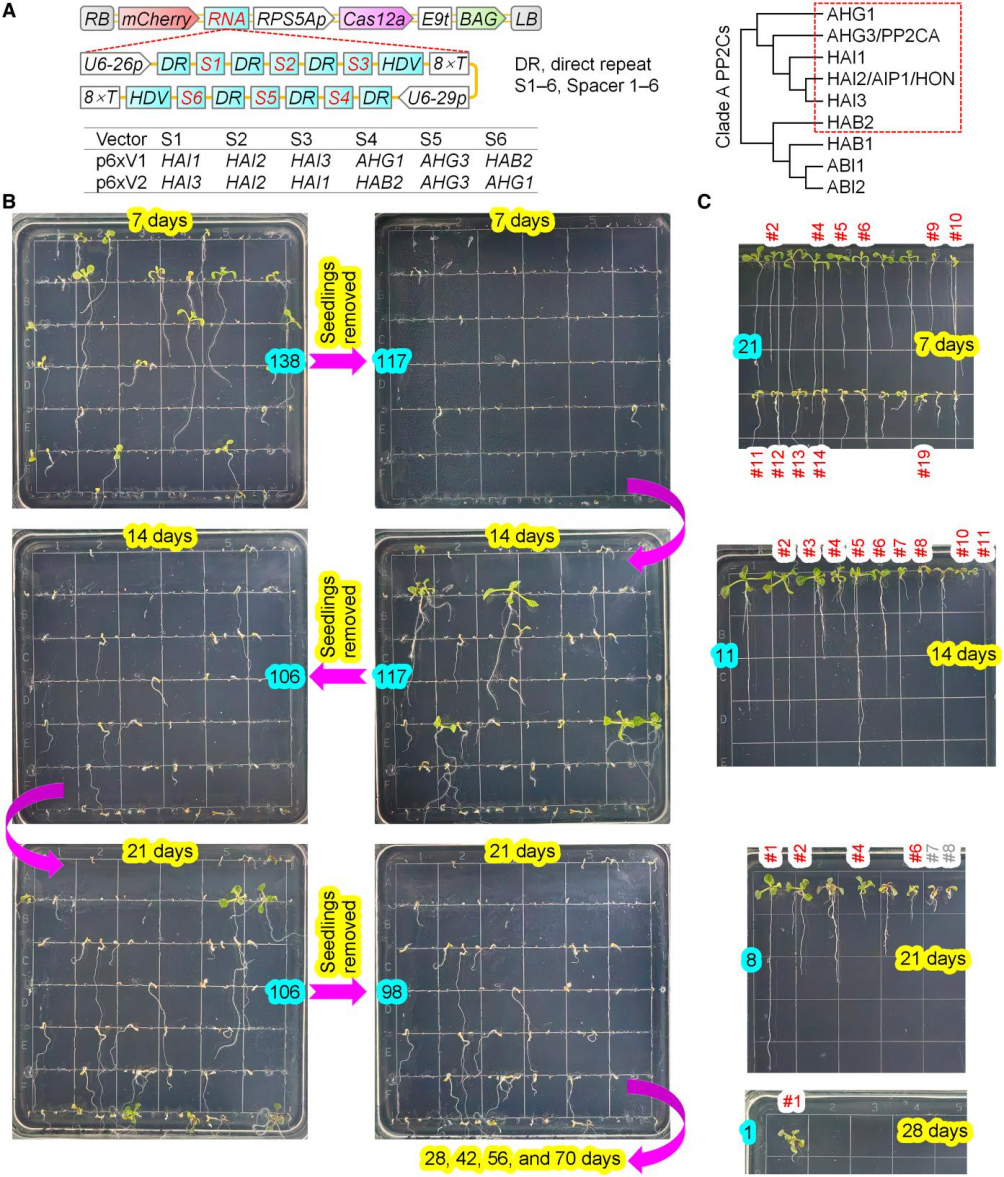
Efficient generation of homozygous sextuple mutants in clade A PP2Cs using the optimized LbCas12a variant. **A)** T-DNA structures of binary vectors and phylogenetic relationships of clade A PP2Cs. DNA elements are annotated as described in Fig. 1A. Phylogenetic tree of 9 clade A PP2C genes is shown, with the 6 targeted genes highlighted (boxed). The same 6 spacers were incorporated into 2 constructs but arranged in different orders within the crRNA arrays. **B)** Delayed germination and growth defects in T1 seeds from p6xV1. T1 transgenic seeds were selected based on red fluorescence, sown on MS medium, vernalized at 4 °C for 3 d, and grown under long-day conditions (16 h light/8 h dark) at 22 °C for the indicated duration. Every 7 d, seedlings were removed to fresh MS plates, with images captured before and after the removal. The numbers highlighted in blue represent numbers of seeds and seedlings in plates. The side length of the squares at the bottom of plates is 1.4 cm. **C)** Seedlings transferred to fresh MS plates were photographed and transplanted to soil after 0 to 7 d of cultivation on MS agar plates. The genotypes were determined by HTS of PCR amplicons. Numbers in red indicate homozygous sextuple mutants, while numbers in gray represent lines that failed to survive after transplantation into soil.

To explore the capability of ttLbUV2 for multiplex genome editing in *Arabidopsis* and generate high-order mutants in clade A PP2Cs for genetic analysis, we generated 2 vectors targeting 6 of 9 clade A PP2Cs (Fig. 2A). The 2 vectors contained identical 6 crRNAs but differed in the arrangement of crRNAs within their tandem arrays, allowing for the assessment of potential positional effects. For efficient crRNA expression, the 6 crRNAs were divided into 2 sets, each consisting of 3 crRNAs, driven separately by the U6-26 or U6-29 promoter (Fig. 2A).

We selected T1 transgenic seeds according to red fluorescence to prevent seeds being omitted from glyphosate-resistant selection since some of the seeds may not germinate due to mutations in multiple clade A PP2Cs. Indeed, the majority of T1 seeds failed to germinate properly even after 70 d of cultivation on MS agar plates: the proportion of seeds displaying impaired germination was 55.8% (77/138) and 51.9% (111/214) for the p6xV1 and p6xV2 vectors, respectively (Fig. 2, B and C; Supplementary Figs. S1 to S3; Table 1). The remaining T1 seeds exhibited either normal or retarded germination. To systematically classify germination patterns, we defined 4 distinct categories based on germination timing and seedling morphology: normal germination (Type-0), seeds germinated and developed into seedlings after cultivation on MS agar plates for 7 d; retarded germination type 1 (Type-1), seeds required 14, 21, or 28 d to germinate and establish seedlings; retarded germination type 2 (Type-2), seeds germinated but exhibited stunted growth, characterized by elongated roots and weak shoot development, requiring >14 d to progress; and retarded germination type 3 (Type-3), seeds failed to germinate even after 70 d of cultivation.

**Table 1.** Efficiency for the generation of homozygous sextuple mutants

Vector	Ratio of mutants to total number of seeds				
	Type-0	Type-1	Type-2	Type-3	Total
p6xV1	52.4% (11/21)	70.0% (14/20)	100.0% (20/20)	100.0% (77/77)	89.9% (124/138)
p6xV2	43.2% (16/37)	33.3% (15/45)	100.0 (21/21)	100.0% (111/111)	80.0% (171/214)

The germination of seeds was categorized into 4 types based on the time taken to germinate and the characteristics of the resulting seedlings: Type-0 (normal germination): seeds germinated after 7 d of cultivation on MS agar. Type-1 (retarded germination): germination occurred after 14, 21, or 28 d. Type-2 (further retarded germination)—germination took 21 d or longer, producing seedlings with long roots and weak buds. Type-3 (no germination): no germination occurred or no seedlings were produced after 70 d of cultivation, and these seeds were predicted to be homozygous sextuple mutants. Two seedlings from p6xV1 and 8 from p6xV2 did not survive after transplantation into soil and were classified as nonsextuple mutants.

High-throughput sequencing (HTS) of PCR amplicons encompassing the target regions revealed that 52.4% (11/21) and 43.2% (16/37) of Type-0 seedlings from p6xV1 and p6xV2, respectively, as well as at least 70.0% (14/20) and 33.3% (15/45) of Type-1 seedlings, were homozygous sextuple mutants (Fig. 2, B and C; Supplementary Figs. S1 to S3; Table 1; Supplementary Table S6). Consequently, the overall proportions of homozygous sextuple mutants among Type-0 and Type-1 seedlings were determined to be 61.0% (25/41) for p6xV1 and 37.8% (31/82) for p6xV2 (Table 1; Supplementary Table S6). To further examine the genotypes of Type-2 T1 plants, we extracted DNA from pooled samples of 20 and 21 Type-2 seedlings from p6xV1 and p6xV2, respectively, and performed HTS of PCR amplicons using the mixed DNA as templates. All HTS reads corresponded exclusively to mutant alleles, with no detectable wild-type sequences, strongly suggesting that all the Type-2 T1 plants were homozygous sextuple mutants (Fig. 2, B and C; Supplementary Figs. S1 to S3; Table 1). Thus, across the 3 types of seedlings, 73.8% (45/61) and 50.5% (52/103) from p6xV1 and p6xV2, respectively, were homozygous sextuple mutants (Table 1).

A detailed mutation analysis revealed that, apart from 2 sextuple mutants (p6xV1-#2 at 21 d and p6xV2-#29 at 7 d), all remaining sextuple mutants harbored at least 1 gene with 1 or 2 alleles carrying insertions or deletions in multiples of 3 nucleotides (3× indels) (Supplementary Table S6). In other words, except for the above 2 cases, no other sextuple mutants were found if excluding those mutant alleles carrying 3× indels (Supplementary Table S6). Alleles harboring the 3× indel mutations may retain at least partial gene functionality, which at least partially explains the variable germination phenotypes of the sextuple mutants. Another possible reason for different germination phenotypes of sextuple mutants including the above 2 is that the mutations in all or a part of 6 genes possibly occurred at the stages of late embryogenesis, germinated seeds, or seedlings. Our results suggest that the mutations, such as some 3× indel mutations, which exert no or weak effects on T1 seed germination, were selectively enriched in the germinated seedlings. Thus, these enrichment effects, along with the observed high efficiency for the generation of homozygous sextuple mutants, make it reasonable to suppose that seeds failing to germinate even after cultivation for 70 d are also likely homozygous sextuple mutants. Accordingly, we estimate the overall editing efficiencies for homozygous sextuple mutant generation to be 89.9% (124/138) for p6xV1 and 80.0% (171/214) for p6xV2 (Table 1). Given the consistently high efficiencies obtained with both p6xV1 and p6xV2, our data suggest that spacer positioning within crRNA arrays exerts a negligible effect on editing efficiency.

### Mutations in the 6 clade A PP2Cs in T1 plants were heritable to the subsequent generation

**Figure 3.**
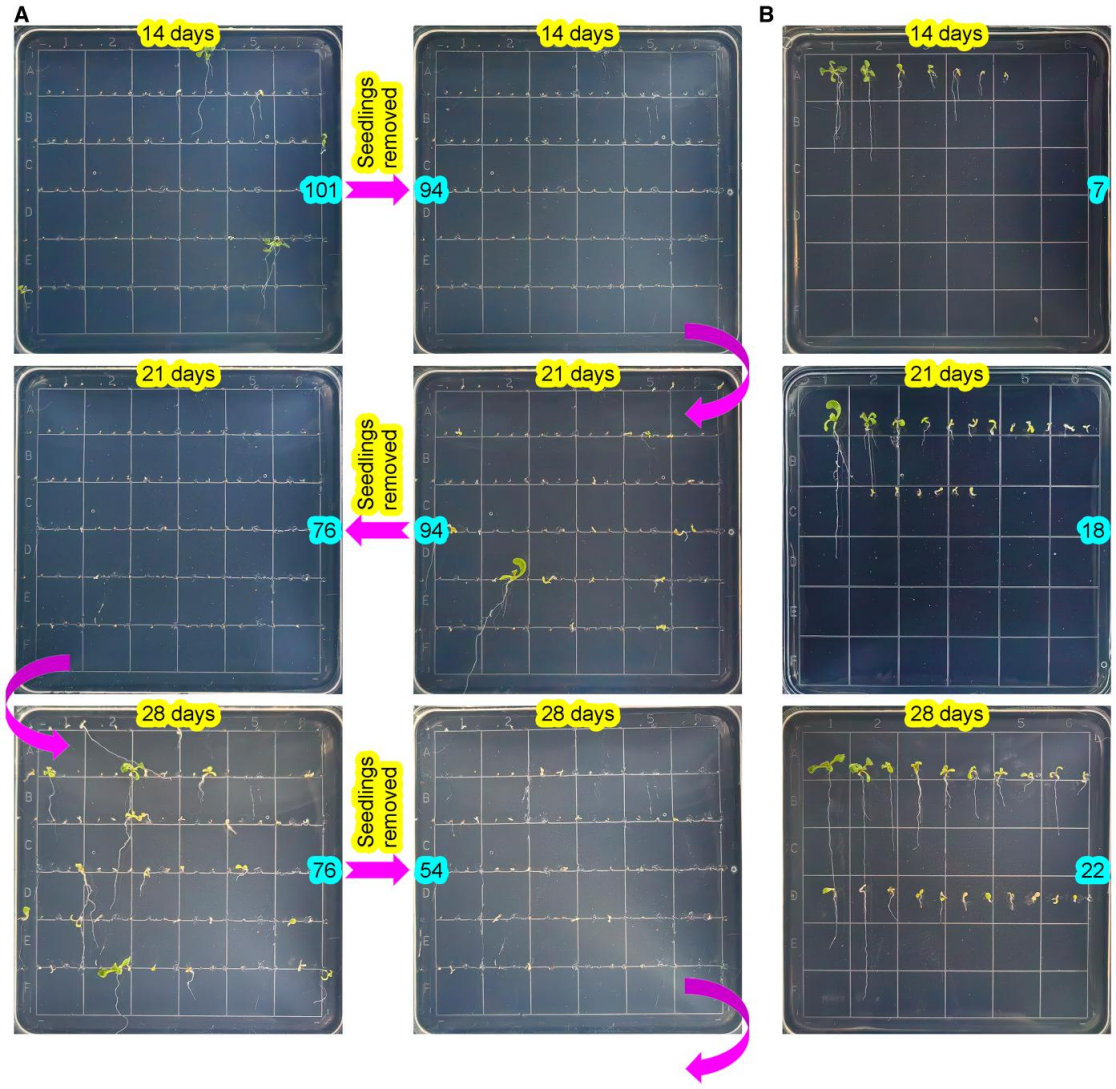
Heritability of mutations in the 6 clade A PP2Cs from T1 to T2 generations. **A)** Delayed germination and growth defects in T-DNA-free T2 seeds from p6xV1-#8. Seedlings were removed to fresh MS plates every 7 d, and pictures were taken before and after the removal. The numbers highlighted in blue represent numbers of seeds and seedlings in plates. The side length of the squares at the bottom of plates is 1.4 cm. **B)** Seedlings transferred to fresh MS plates were photographed and subsequently transplanted to soil after 0 to 7 d of cultivation on MS agar plates. The numbers of seeds and/or seedlings are indicated. Genotyping of soil-grown seedlings confirmed the heritability of mutations in the 6 clade A PP2Cs.

To test the heritability of mutations in the 6 clade A PP2Cs, we isolated T-DNA-free T2 seeds from 3 p6xV1 and 3 p6xV2 T1 transgenic lines, identified as homozygous or biallelic sextuple mutants. As anticipated, some of the T-DNA-free T2 seeds displayed retarded germination (Fig. 3; Table 2). HTS of some seedlings derived from seeds with normal or retarded germination indicated that, except for p6xV2-#5-1, all analyzed T-DNA-free T2 plants were homozygous sextuple mutants (Supplementary Table S7). These results demonstrate that mutations in the 6 clade A PP2Cs in T1 plants are heritable to the subsequent generation. T-DNA-free T2 plants and their T1 lines harbored the same indel mutations (Supplementary Table S7), which provides additional evidence that the mutations in T1 plants are stably transmitted to T2 plants.

**Table 2.** Germination of T-DNA-free T2 seeds

Vector	T1	T-DNA-free T2 seeds		
		Type-0	Type-1 or Type-2	Type-3
p6xV1	#2	53.7% (22/41)	43.9% (18/41)	2.4% (1/41)
	#11	20.0% (16/80)	58.8% (47/80)	21.2% (17/80)
	#14	78.6% (22/28)	0.0% (0/28)	21.4% (6/28)
p6xV2	#5	52.3% (34/65)	32.3 (21/65)	15.4% (10/65)
	#8	0.0% (0/101)	65.3% (66/101)	34.7% (35/101)
	#27	67.8% (80/118)	19.5% (23/118)	12.7% (15/118)

Seed germination types (Type-0 to Type-3) are described in Table 1. Germination was assessed over 7, 14, 28, and 42 d of cultivation. However, for sample #5, data were collected at 28 d due to contamination of the culture plate before the 42-d period.

Genotypic analysis revealed that all sextuple mutants contained at least 1 gene with 1 or 2 alleles carrying 3× indel mutations (Supplementary Table S7). Notably, when sextuple mutants harboring such alleles were excluded, no Type-0 or Type-1 sextuple mutants were observed (Supplementary Table S7). Within the T2 sextuple mutants, #8-12 and #8-17 (derived from p6xV2-#8) harbored loss-of-function mutations in 5 genes, with *HAI1* carrying a 12 bp deletion mutation, suggesting that the presence of a partially functional *HAI1* allele alone does not entirely inhibit seed germination. Collectively, these results reinforce the above observations in T1 plants that germination of sextuple mutant seeds is completely disrupted if all 6 genes harbor loss-of-function mutations.

### Comparison of ttLbUV2 with other LbCas12a variants in editing efficiencies

**Figure 4.**
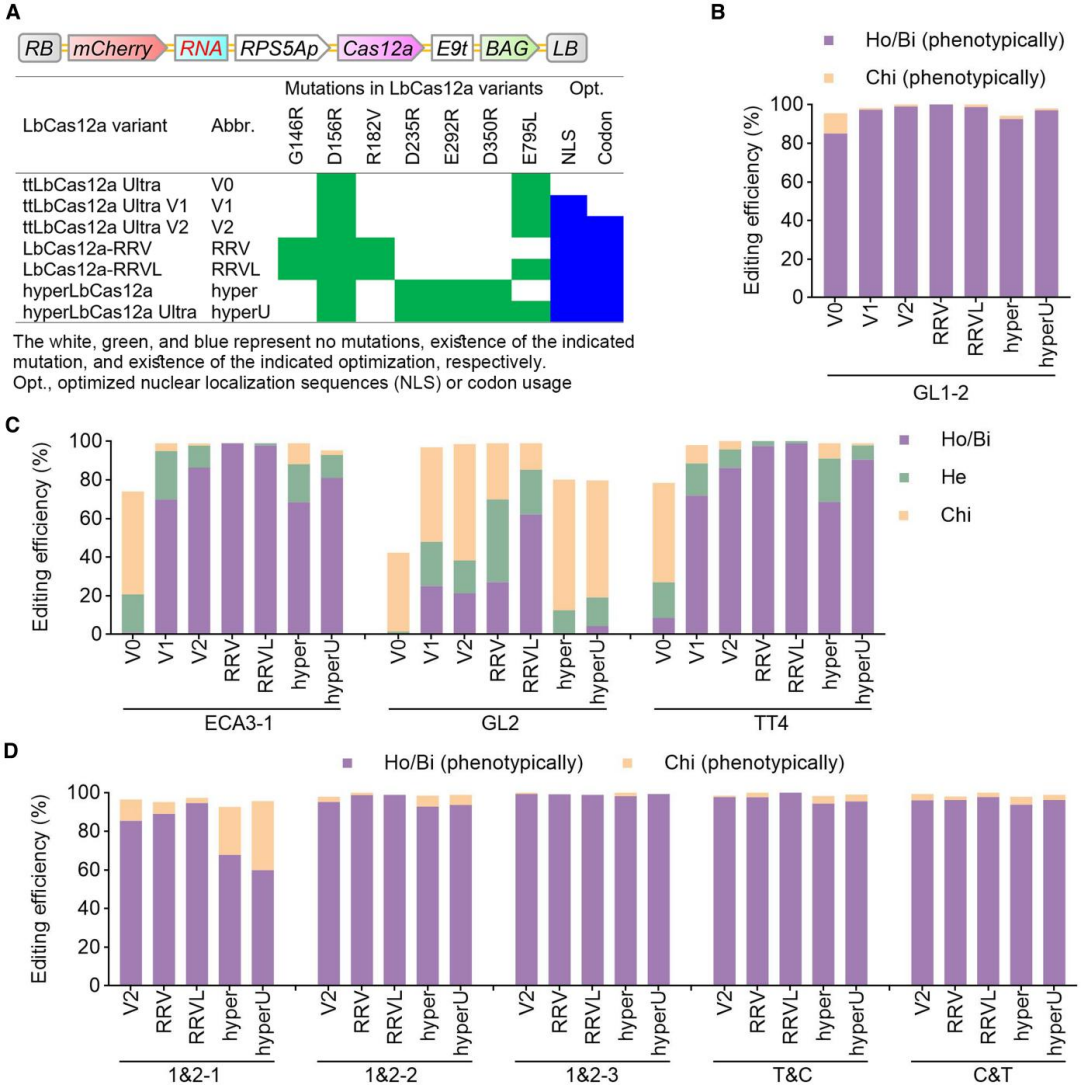
Comparative editing efficiencies of 7 LbCas12a variants across single and dual targets. **A)** T-DNA structures of binary vectors. For details on the DNA elements and crRNA expression cassettes, refer to Fig. 1A. Mutations in LbCas12a variants, along with optimization of NLS sequences and codon usage, are indicated. Variants V0–V2 share the same mutations but differ in NLS sequences and/or codon usage. **B** to **D)** Editing efficiencies of 7 LbCas12a variants across single and dual targets from 1,482 **B)**, 2,025 **C)**, or 3,176 **D)** T1 transgenic lines. The labels below the *x* axes **D)** represent the spacers described in Fig. 1. HTS was used to assess editing efficiencies at ECA3-1, GL2, and TT4, while phenotypic analysis was performed for other targets. Ho/Bi, homozygous or biallelic; Chi, chimeric. Data for variants V0 and V2 at the 4 single targets were previously reported (Xin et al. 2024).

In our previous report, we demonstrated that the ttLbUV2 variant, which incorporates optimized NLS sequences and codon usage, significantly enhanced genome-editing efficiency compared with the original ttLbCas12a Ultra variant (ttLbUV0). In this report, to determine which of the 2 factors is decisive for the improvement, we generated another ttLbCas12a Ultra variant—ttLbCas12a Ultra V1 (ttLbUV1)—which encodes the same protein sequence as ttLbUV2 but has different codon usage (Fig. 4A). The ttLbUV1 variant was derived from ttLbUV0, and thus both maintain the same codon usage (Fig. 4A). To further benchmark ttLbUV2 against other highly active variants, we developed 4 additional variants, including LbCas12a-RRV (RRV), LbCas12a-RRVL (RRVL), hyperCas12a (hyper), and hyperCas12a Ultra (hyperU) (Fig. 4A). These 4 variants all possess the same NLS sequences and codon usage as ttLbUV2.

Compared to ttLbUV0, ttLbUV1 and ttLbUV2 improved editing efficiencies across all the 4 tested targets and markedly improved editing efficiencies at the ECA3-1, GL2, and TT4 targets, where ttLbUV0 exhibited low editing efficiencies (Fig. 4, B and C; Supplementary Table S8). Further comparisons between ttLbUV1 and ttLbUV2 revealed that ttLbUV1 maintained similar editing efficiency at the GL1-2 target, exhibited slightly lower editing efficiencies for homozygous mutations at the ECA3-1 and TT4 targets, and achieved slightly higher editing efficiency for homozygous mutations at the GL2 target (Fig. 4, B and C; Supplementary Table S8). These results demonstrate that optimized NLS sequences rather than codon usage are the decisive reason for the high efficiency of ttLbUV2.

Relative to ttLbUV2, RRV and RRVL exhibited similarly high editing efficiencies at all 12 targets, with RRVL—which carries an additional E795L mutation—showing slightly superior performance over RRV (Fig. 4, B to D; Supplementary Tables S2, S3, and S8). Likewise, both the hyper and hyperU variants displayed comparable editing efficiencies to ttLbUV2 at nearly all targets, except for GL2, and the incorporation of the E795L mutation in hyperU resulted in a modest enhancement in editing efficiencies across all 12 targets but CHLI1&2-1 (Fig. 4, B to D; Supplementary Tables S2, S3, and S8). Overall, these results demonstrate that although RRVL exhibits the highest editing efficiency, ttLbUV2, which contains only 2 amino acid substitutions, maintains sufficiently high editing efficiencies for practical applications.

### Development of tools based on a Cas12i3 and 2 AsCas12f variants for genome editing in *Arabidopsis*

**Figure 5.**
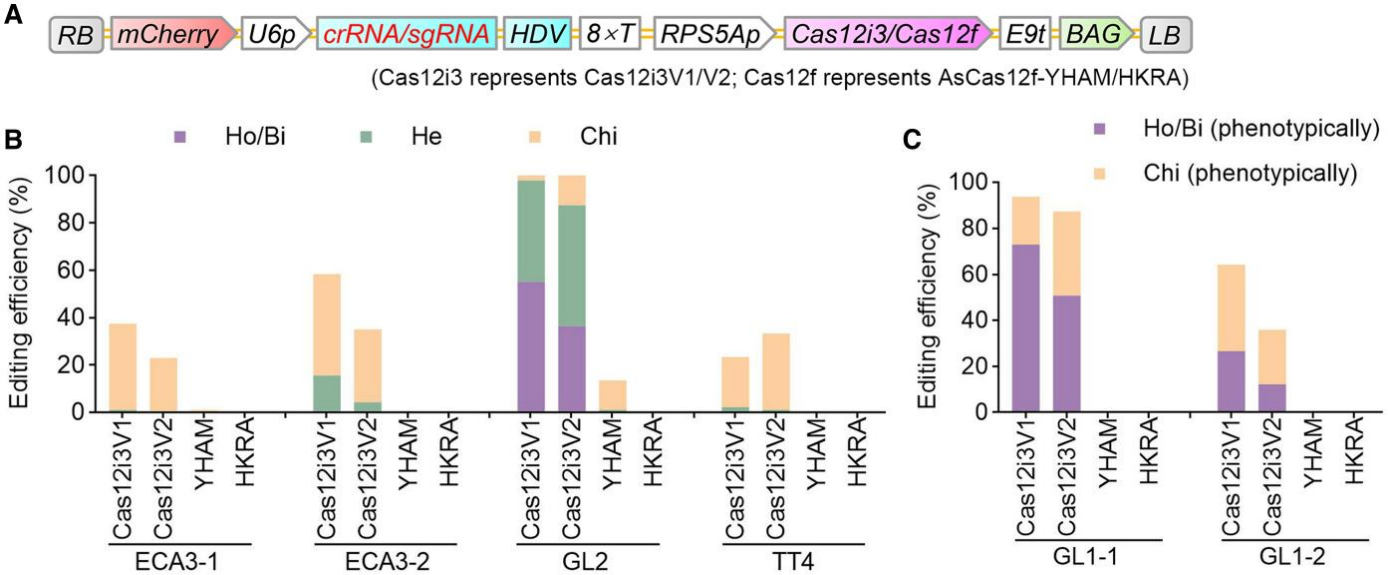
Editing efficiencies of Cas12i3 or AsCas12f variants across 6 single targets. **A)** T-DNA structures of binary vectors with DNA elements and crRNA expression cassettes annotated as described in Fig. 1A. Cas12i3V1 and Cas12i3V2 share identical mutations but differ in codon usage. **B** and **C)** Editing efficiencies of the variants across 6 single targets from 1,530 **B)** and 1,014 **C)** T1 transgenic lines. Editing efficiencies were determined by HTS for ECA3-1, ECA3-2, GL2, and TT4 and by phenotypic analysis for targets GL1-1 and GL1-2. Ho/Bi, homozygous or biallelic; He, heterozygous; Chi, chimeric.

To develop tools based on Cas12i3 and AsCas12f variants for genome editing in *Arabidopsis*, we constructed 4 vectors harboring Cas12i3V1, Cas12i3V2, AsCas12f-YHAM, and AsCas12f-HKRA (Fig. 5A). All variants were designed with identical NLS sequences at both termini, and the Cas12i3V1 variant was codon-optimized for monocots, whereas the other 3 variants were codon-optimized for dicot plants. Cas12i3V1 and Cas12i3V2 variants harbor the S7R, D233R, D267R, N369R, and S433R mutations, corresponding to previously reported variants but with different NLS sequences and codon usage. For the 2 AsCas12f variants, we used an optimized sgRNA scaffold (sgRNA_DS3–5_v7) (Hino et al. 2023; Ye et al. 2024).

Analysis of mutations in T1 transgenic plants indicated that the 2 Cas12i3 variants exhibited relatively high editing efficiencies for heritable (homozygous or heterozygous) mutations at GL2, GL1-1, GL1-2, and ECA3-2 targets and very low editing efficiencies at the remaining 2 targets (Fig. 5, B and C; Supplementary Table S9). Unexpectedly, Cas12i3V1 with optimized codons for monocots outperformed Cas12i3V2 with optimized codons for dicots, suggesting that besides optimization of codon usage, additional sequence refinements are necessary to maximize editing efficiency. Our results demonstrate that Cas12i3V1 is a viable candidate for practical genome-editing applications in *Arabidopsis*. Surprisingly, we did not achieve acceptable editing efficiencies for the 2 AsCas12f variants at all 6 targets, with AsCas12f-YHAM inducing only weak chimeric mutations at the GL2 target (Fig. 5, B and C; Supplementary Table S9). These findings underscore that further optimizations are required for applications of the 2 AsCas12f variants in *Arabidopsis*.

## Discussion

In this report, we present a comprehensive evaluation of ttLbUV2, an optimized LbCas12a variant, demonstrating its high editing efficiency and low target bias in *Arabidopsis*. We provide empirical evidence that ttLbUV2 is particularly effective in generating sextuple mutants in clade A PP2Cs. Through a comparative analysis of 7 LbCas12a variants, we identify optimized NLS sequences, rather than codon usage, as the primary factor contributing to ttLbUV2's enhanced efficiency, and the E795L mutation has synergistic effects on other mutations in some highly efficient variants, such as LbCas12a-RRV and hyperCas12a. Furthermore, we investigate the editing efficiencies of a Cas12i3 variant and 2 AsCas12f variants, demonstrating that the Cas12i3 variant with monocot-preferred codons, Cas12i3V1, is sufficiently high for genome editing in *Arabidopsis*, although the issue of relatively high target bias needs to be addressed.

Low target bias is a critical parameter for the evaluation of a CRISPR/Cas tool. Ideally, low target bias of a genome editing tool means that it is always highly efficient across almost all targets selected randomly or by some known rules besides PAM (Concordet and Haeussler 2018; Kim et al. 2018). Tools with minimal target bias obviate the need for extensive target validation, thereby significantly enhancing research efficiency and minimizing time, labor, and financial costs, particularly in multiplex genome editing. Previously, we demonstrated that the optimized LbCas12a variant, ttLbUV2, was highly efficient for the 4 reported targets (Xin et al. 2024). In this report, we expanded our analysis to 18 additional targets in T1 transgenic plants (Fig. 1, Supplementary Tables S1 to S3) and confirmed consistently high editing efficiencies of ttLbUV2 across all tested targets. We also achieved sextuple mutants in clade A PP2Cs with remarkable efficiency. None of the 24 targets in total underwent prior validation before being used for the generation of transgenic plants, which demonstrated that the optimized LbCas12a variant possesses exceptionally low target bias in *Arabidopsis* genome editing.

A previous study reported that an optimized CRISPR/Cas9 vector targeting 7 genes, including *GL1*, *SMB*, *ARF7*, *ARF19*, *ADH1*, *FLS2*, and *EFR*, achieved 28.0%, 37.0%, and 5.0% editing efficiencies for the generation of T-DNA-free T2 quintuple, sextuple, and septuple mutants, respectively (Develtere et al. 2024). This was previously recognized as the highest multiplex genome-editing efficiency reported in *Arabidopsis*. In this paper, we achieved nearly 100% editing efficiency for the generation of T-DNA-free T2 sextuple mutants, which has at least broken the record for the highest editing efficiency for the generation of sextuple mutants. In addition, we achieved as high as 73.8% (45/61) editing efficiency, based on available data, for the generation of T1 sextuple mutants without wild-type alleles and predicted that it could be as high as 89.9% (124/138) if those never-germinating seeds were considered (Fig. 2; Supplementary Figs. S1 to S3; Table 1; Supplementary Table S6). Thus, these results position the optimized LbCas12a variant as the most effective tool for multiplex genome editing in *Arabidopsis*. Beyond its unparalleled efficiency, the system boasts additional advantages, such as simplified guide RNA assembly and low target bias, further enhancing its utility for multiplex genome-editing applications.

In *Arabidopsis*, type 2C protein phosphatases (PP2Cs) from clade A act as negative regulators of the ABA pathway through their interaction with SnRK2s, and this negative regulation is unbraked by ABA receptors from the PYR/PYL/RCAR family in an ABA-dependent manner (Zhao et al. 2018). A duodecuple mutant carrying mutations in 14 ABA receptor genes was constructed using multiple rounds of CRISPR/Cas9-mediated genome editing and a cotransformation strategy, uncovering the role of ABA receptors in suppressing ABA-independent SnRK2 activity (Zhao et al. 2018). Additionally, a decuple mutant encompassing all 10 members of the SnRK2 kinase family, constructed via sequential crossing of T-DNA insertion mutants, demonstrated the essential roles of SnRK2 kinases in osmotic stress responses in vivo (Fujii et al. 2011). However, high-order mutants in 9 PP2C clade A genes in the ABA signaling pathway have not yet been established, although 2 quadruple mutants (*abi1 hab1 ahg3 hai1* and *abi1 hab1 ahg3 abi2*) and 2 triple mutants (*abi1 hab1 ahg3* and *abi1 hab1 abi2*) were generated (Rubio et al. 2009; Antoni et al. 2012; Rodrigues et al. 2013).

In this study, we efficiently generated sextuple mutants in 9 clade A PP2Cs using the optimized LbCas12a variant, and our results suggest that loss-of-function mutations in the 6 genes, including *AHG1*, *AHG3*, *HAI1*, *HAI2*, *HAI3*, and *HAB2*, will lead to the production of seemingly never-germinating seeds. There are 6 combinations of the 6 genes in generation of quintuple mutants and 2 quintuple mutants, p6xV2-#8-12 and 17, carrying no loss-of-function mutations in *HAI1*, exhibited delayed seed germination (Fig. 3; Table 2; Supplementary Table S7). It remains unknown whether the other 5 quintuple mutant seeds will exhibit seemingly never-germinating phenotypes. It will be easy to generate these 5 quintuple mutants to test seed germination phenotypes using the vectors and targets validated in this study. Furthermore, constructing a comprehensive library of clade A PP2C mutant combinations and evaluating their impacts on seed germination and agronomic traits could provide critical insights for breeding crop varieties with enhanced resistance to preharvest sprouting and improved storage longevity.

Comparisons of LbCas12a-RRV (G146R, D156R, and R182V) and hyperCas12a (D156R, D235R, E292R, and D350R) with their variants harboring the E795L mutation, LbCas12a-RRVL and hyperCas12a Ultra, respectively, indicated that the E795L mutation had synergistic effects on the enhancement of these 2 or other variants (Fig. 4). In a previous report, compared to LbCas12a-RRV, LbCas12a-RRVQ with E795Q mutation showed compromised editing efficiency in human, rice, and tomato cells or protoplasts (Zhang et al. 2023). LbCas12a-RRV also outperformed LbCas12a-RRVQ in stable transgenic poplar (Zhang et al. 2023). These results suggest that while E795Q alone enhances activity beyond that of E795L, the E795L mutation confers a greater synergistic effect when combined with other optimized variants. It was indicated that LbCas12a-RRV functioned not only at canonical TTTV PAM sites but also noncanonical VTTV PAM sites. In the future, it is interesting to compare LbCas12a-RRVL with LbCas12a-RRV in editing efficiencies at noncanonical VTTV PAM sites. Although LbCas12a-RRVL and LbCas12a-RRV are the 2 most efficient variants, ttLbUV2 harboring only 2 mutations (D156R and E795L) has showed sufficiently high editing efficiencies for generation of single, double, or sextuple mutants. As the simplest variant with sufficiently high editing efficiency, the ttLbUV2 variant has 2 advantages. One is that ttLbUV2 is supposed to have lower editing efficiency at noncanonical VTTV PAM sites than LbCas12a-RRV/RRVL and thus may have fewer potential off-target sites. Another is that ttLbUV1 or ttLbUV2 may help generate highly active LbCas12a variants with altered PAM specificities by combining mutations from ttLbUV2 with those from the improved LbCas12a variants with altered PAM specificities, such as impLbCas12a harboring D156R, G532R, K538V, Y542R, and K595R mutations (Toth et al. 2020). The use of variants with fewer mutations will streamline the optimization process and make it more predictable.

The hyperCas12a variant was developed through structure-guided protein engineering and based on the rational design to increase the binding affinity of Cas12a to its negatively charged target DNA (Guo et al. 2022). The LbCas12a-RRV, LbCas12a-RRVL, and ttLbUV2 variants outperformed hyperCas12a and hyperCas12a Ultra, suggesting that increasing the binding affinity does not necessarily associate with improved cleavage activity. Although the hyperCas12a Ultra variant harbored both mutations from ttLbUV2, hyperCas12a Ultra did not outperform ttLbUV2, suggesting that the 3 mutations specific to hyperCas12a exert negative effects on the enhancement of the E795L mutation.

Relative to ttLbUV2, the Cas12i3 variant also maintains high efficiency but exhibits a notable target bias (Fig. 5). Although the more permissive PAM of the Cas12i3 variant may compensate for its issue of target bias to an extent, further optimizations of Cas12i3 activity along with deep learning-based prediction of crRNA activity are required to address this issue. Optimization of crRNA expression with the composite promoter, along with fusion of the Cas12i3 variant with appropriate exonucleases, will also help address the issue (Wang et al. 2024, 2025).

The 2 AsCas12f variants displayed extremely low editing efficiency or severe issue of target bias in *Arabidopsis* (Fig. 5). We propose 2 potential explanations for this observation. First, the NLS sequence modifications that successfully enhanced other Cas12a variants may not be suitable for the 2 AsCas12f variants, potentially affecting their nuclear transport and functionality. Second, the 2 AsCas12f variants may be highly sensitive to relatively low temperature, with their activity suppressed under the standard 22 °C growth conditions used for *Arabidopsis*.

Collectively, our findings establish the LbCas12a variant harboring D156R and E795L mutations as the most effective tool for achieving high-efficiency multiplex genome editing in *Arabidopsis*. The optimized LbCas12a variant successfully generated seemingly never-germinating seeds carrying mutations in 6 of 9 clade A PP2Cs and an editing efficiency of at least 73.8% (45/61) was achieved for the generation of T1 homozygous sextuple mutants, demonstrating its robustness in multiplex genome editing in *Arabidopsis*. Additionally, our results highlight the critical role of NLS sequence optimization in enhancing Cas12a-mediated editing efficiency. While the Cas12i3 variant also exhibits high editing efficiency in *Arabidopsis*, further optimizations are necessary to mitigate its relatively high target bias, ensuring broader applicability in plant genome editing in the future.

## Materials and methods

### Vector construction

All primers used in this study are listed in Supplementary Table S10, and the sequences of all target sites are listed in Supplementary Tables S11 and S12. Vectors described in this study are listed in Supplementary Table S13 and available from Addgene (#231386 and #231387 for pBG-ttLbUV2 and pAGC-Lb12-P1Bb, respectively). The sequences of Cas12 variants, the cloning cassettes for the assembly of Cas12a/i3 crRNAs or Cas12f sgRNAs, and the final expression cassettes are provided in Supplementary Sequences S1.

We amplified a fragment with ttLbUV0-BsF and ttLbUV0-BsR from pBG-ttLbU (Xin et al. 2024), purified the fragment, digested it with *Bsa*I, and inserted the digested fragment into the BsaI sites of a synthetic fragment, resulting in the generation of pL2R4-ttLbUV1. We digested pL2R4-ttLbUV1 with *Xba*I and *Sac*I and inserted the digested fragment into the *Xba*I and *Sac*I sites of pBG-ttLbU, resulting in the generation of pBG-ttLbUV1.

We amplified 2 PCR fragments from pBG-ttLbUV2 (Xin et al. 2024) with PstI-upF and L795E-BsR and L795E-BsF and tE9-IDR2 primer pairs. We mixed the 2 PCR fragments, purified them, digested them with *Bsa*I, and ligated it with a blunt-end cloning vector, resulting in the generation of pCBC-L795E. We ligated *Pst*I and *Sac*I-digested pCBC-L795E with PstI and SacI-digested pUC57-hyperU-5 harboring a synthetic fragment, resulting in the generation of pUC57-hyperLb12. We ligated *Pst*I and *Sac*I-digested pUC57-ttLbUV2 with *Pst*I and *Sac*I-digested pUC57-hyperU-5, resulting in the generation of pUC57-hyperLb12U. We ligated *Xba*I and *Sac*I-digested pUC57-hyperLb12 or pUC57-hyperLb12U with *Xba*I and *Sac*I-digested pBG-ttLbUV2, resulting in the generation of pBG-hyperLb12 or pBG-hyperLb12U, respectively.

We amplified 3 PCR fragments from pBG-ttLbUV2 with RPS5A-IDF2 and G146R-R, G146R-F and R182V-R, and R182V-F and PstI-dnR primer pairs. We performed second round of PCRs using the 3 PCR fragments as templates and primers RPS5A-IDF2 and PstI-dnR. We purified the PCR fragment and ligated it with a blunt-end cloning vector, resulting in the generation of pCBC-RRV. We digested pCBC-RRV with *Xba*I and *Pst*I and inserted the digested fragment into *Xba*I and *Pst*I sites of pUC57-hyperLb12 or pUC57-hyperLb12U, resulting in the generation of pUC57-LbCas12a-RRV and pUC57-LbCas12a-RRVL. We ligated *Xba*I and *Sac*I-digested pUC57-LbCas12a-RRV or pUC57-LbCas12a-RRVL with *Xba*I and *Sac*I-digested pBG-ttLbUV2, resulting in the generation of pBG-Lb12-RRV or pBG-Lb12-RRVL, respectively.

We amplified a fragment with primers pAGC-F and pAGC-R from a pUC57-derived vector, where the *Bsa*I site was disrupted, and another fragment with primers iCeuI-EcF and iSceI-EcR from pUC57-8xLb-P1 harboring a synthetic fragment. We mixed the 2 fragments, purified them, digested them with *Eco*RV, and ligated them, resulting in the generation of pABC-8xLb-P1.

We amplified 2 PCR fragments from pBG-ttLbUV2 with a primer pair (U6-HiF2 and U6-R) and a combination of 4 primers (U6-i3-F, U6-i3-F01, U6-i3-F02, and U6t-SpeR). We performed second round of PCR reactions using the 2 PCR fragments as templates and primers U6-HiF2 and U6t-SpeR. We purified the PCR fragment, digested it with *Hin*dIII and *Spe*I, and ligated the digested fragment with *Hin*dIII and *Spe*I-digested pBG-ttLbUV2, resulting in the generation of pBG-Cas12i3-P1. We replaced the *Xba*I-*Sac*I fragment of pBG-Cas12i3-P1 with synthetic Cas12i3V1 or Cas12i3V2 digested with *Xba*I and *Sac*I, resulting in the generation of pBG-Cas12i3V1 or pBG-Cas12i3V2, respectively.

We amplified a fragment from pHEE401E with primers SpR-BsF and SpR-BsR, purified the fragment, digested it with *Hin*dIII and *Spe*I, and ligated with the *Hin*dIII and *Spe*I-digested pBG-ttLbUV2, resulting in the generation of pBG-AsCas12f-P1. We replaced the *Xba*I-*Sac*I fragment of pBG-AsCas12f-P1 with *Xba*I and *Sac*I-digested synthetic AsCas12f-YHAM or AsCas12f-HKRA, resulting in the generation of pBG-YHAM or pBG-HKRA, respectively.

To generate final vectors harboring 1 crRNA, we prepared an insert by annealing 2 primers for each crRNA and used the insert to replace the *Bsa*I-*Bsa*I fragments of the Cas12 binary vectors (Supplementary Tables S11 to S13; Supplementary Sequences S1). To generate final vectors harboring 2 crRNAs, we prepared 2 inserts by annealing 2 primers for each insert and used the 2 inserts to replace the *Bsa*I-*Bsa*I fragments of the Cas12 binary vectors (Supplementary Tables S11 and S13; Supplementary Sequences S1).

To generate final vectors harboring 6 crRNAs, we prepared 6 inserts by annealing 2 primers for each insert. We used the 6 inserts and pAGC-Lb12-P1Bb to set up *Bbs*I-based Golden Gate reactions, resulting in the generation of pAGC-S1–S6. We used pAGC-S1–S6 and pBG-ttLbUV2 to set up *Bsa*I-based Golden Gate reactions, resulting in the generation of 2 final LbCas12 binary vectors targeting the 6 clade A PP2Cs (Supplementary Tables S11 and S13; Supplementary Sequences S1).

### Generation of transgenic plants and analysis of mutations

We introduced the 81 vectors generated in this report (Supplementary Table S13) individually into *Agrobacterium* strain GV3101. We transformed the *Arabidopsis thaliana* accession Col-0 via the floral dip method. Primary (T_1_) transformants were selected on Murashige and Skoog (MS) medium containing 100 *μ*M glyphosate before transferring the resistant seedlings to soil. For p6xV1 and p6xV2, primary (T_1_) transformants were selected by red fluorescence of transgenic seeds.

For the vectors targeting *GL1*, *CHLI1* and *CHLI2*, and *TRY* and *CPC*, we analyzed the editing efficiency by counting the number of plants with expected phenotypes. For the other targets, we analyzed the editing efficiency by HTS. HTS-based mutation analysis involved PCR amplification of DNA fragments flanking the target sites using primers listed in Supplementary Table S10, followed by sequencing and analysis via the Hi-Tom platform (Liu et al. 2019b). Mutation efficiency was calculated as the ratio of the number of plants with detectable mutations to the total number of transgenic plants. When ≥95% HTS reads from a line represented the same type of mutation, we scored this line as a homozygous (Ho) mutant. When ≥95% HTS reads from a given line represented more than 1 type of mutation, we considered this line biallelic (Bi). For nonhomozygous and nonbiallelic mutants, when ≥45% or <45% HTS reads from a line represented 1 type of mutation, we scored this line as heterozygous (He) or chimeric (Chi), respectively.

For analysis of off-target mutations, we used primers listed in Supplementary Table S10 to amplify PCR fragments spanning the potential off-target sites, and the resulting PCR products were analyzed by Sanger sequencing to detect unintended mutations.

### Analysis of heritable mutations

To analyze heritable mutations, we selected T2 seeds lacking red fluorescence under a stereo fluorescence microscope (Gao et al. 2016) and then analyzed the presence of mutations in these T-DNA-free T2 plants. Heritable mutations in *TRY* and *CPC* were identified based on clustered trichome phenotypes, while those in the 6 clade A PP2Cs were quantified through HTS.

### Accession numbers

The genes used in this study and their accession numbers are as follows: *GL1*, At3G27920; *GL2*, At1G79840; *TT4*, At5G13930; *ECA3*, At1G10130; *CHLI1*, At4g18480; *CHLI2*, At5g45930; *CPC*, At2G46410; *TRY*, At5G53200; *HAI1*, At5G59220; *HAI2*, At1g07430; *HAI3*, At2g29380; *PP2CA*, At3g11410; *AHG1*, At5g51760; and *HAB2*, At1g17550.

## Supplementary Material

kiaf315_Supplementary_Data

## Data Availability

The data underlying this article will be shared on reasonable request to the corresponding author.

## Supplementary data

The following materials are available in the online version of this article.

**Supplementary Figure S1.** Germination delay and growth defects of T1 seeds from p6xV1 after 28, 42, 56, and 70 d of cultivation.

**Supplementary Figure S2.** The optimized LbCas12a variant efficiently generates homozygous sextuple mutants in clade A *PP2C* genes.

**Supplementary Figure S3.** Germination delay and growth defects of T1 seeds from p6xV2 after 28, 42, 56, and 70 d of cultivation.

**Supplementary Table S1.** Editing efficiencies of *ttLbUV2* across 12 *GL1* target sites.

**Supplementary Table S2.** Editing efficiencies of 5 Cas12a variants in generating *chli1 chli2* double mutants.

**Supplementary Table S3.** Editing efficiencies of 5 Cas12a variants in generating t*ry cpc* double mutants.

**Supplementary Table S4.** Analysis of mutations in *TRY* and *CPC* in T-DNA-free T2 plants.

**Supplementary Table S5.** Analysis of off-target mutations.

**Supplementary Table S6.** Mutations in the 6 clade A *PP2Cs* in T1 transgenic lines.

**Supplementary Table S7.** Mutations in the 6 clade A *PP2Cs* in T-DNA-free T2 plants.

**Supplementary Table S8.** Editing efficiencies of 7 LbCas12a variants across 4 target sites.

**Supplementary Table S9.** Editing efficiencies of 1 Cas12i3 variant and 2 Cas12f variants across 6 target sites.

**Supplementary Table S10.** Primer sequences used in this study.

**Supplementary Table S11.** Sequences of target sites for LbCas12a or Cas12i3.

**Supplementary Table S12.** Sequences of target sites for Cas12f variants.

**Supplementary Table S13.** List of binary vectors used in this study.

**Supplementary Sequences S1.** Sequences of Cas12 variants and crRNA/sgRNA cassettes.

## Dive Curated Terms

The following phenotypic, genotypic, and functional terms are of significance to the work described in this paper:

CPC Gramene: AT2G46410
CPC Araport: AT2G46410

CHLI1 Gramene: AT4G18480
CHLI1 Araport: AT4G18480

AHG1 Gramene: AT5G51760
AHG1 Araport: AT5G51760

HAB2 Gramene: AT1G17550
HAB2 Araport: AT1G17550

TT4 Gramene: AT5G13930
TT4 Araport: AT5G13930

TRY Gramene: AT5G53200
TRY Araport: AT5G53200

CHLI2 Gramene: Cre12.g510800
CHLI2 Araport: Cre12.g510800

ECA3 Gramene: AT1G10130
ECA3 Araport: AT1G10130

GL2 Gramene: AT1G79840
GL2 Araport: AT1G79840

PP2CA Gramene: AT3G11410
PP2CA Araport: AT3G11410

GL1 Gramene: AT3G27920
GL1 Araport: AT3G27920

HAI1 Gramene: At5G59220
HAI1 Araport: At5G59220

HAI2 Gramene: At1g07430
HAI2 Araport: At1g07430

HAI3 Gramene: At2g29380
HAI3 Araport: At2g29380
