# Supplementary material for: Never-germinating Arabidopsis seeds with LbCas12a-induced mutations in 6 clade A type 2C protein phosphatase genes
Source: Plant Physiol. 2025 Jul 17;198(3):kiaf315. doi: 10.1093/plphys/kiaf315 (PMC12301882; doi:10.1093/plphys/kiaf315)
Supplement: kiaf315_Supplementary_Data [file kiaf315_supplementary_data.zip › Xin et al. 2025 PP R2 Supplementary Data.pdf]

Supplementary Data

Never-Germinating Arabidopsis Seeds with LbCas12a-Induced Mutations in Six Clade A Type 2C Protein Phosphatase Genes

Cuiping **Xin**,[1] Yu **Lu**,[1] Syeda Leeda **Gul**,[1] Wei **Sun**,[1] Zhenghong **Cao**,[1] Xiangchao **Kong**,[1] Kexin **Fan**,[1] Siyun **Li**,[1] Xiaohan **Liu**,[1] Xue-Chen **Wang**,[1] Qi-Jun **Chen**[1,2,*]

[1]State Key Laboratory of Plant Environmental Resilience, College of Biological Sciences, China Agricultural University, Beijing 100193, China

[2]Center for Crop Functional Genomics and Molecular Breeding, China Agricultural University, Beijing 100193, China

[*] Corresponding author: qjchen@cau.edu.cn

Table of contents

Supplementary Figure S1. Germination delay and growth defects of T1 seeds from p6xV1 3

Supplementary Figure S2. The optimized LbCas12a variant efficiently generates 4

Supplementary Figure S3. Germination delay and growth defects of T1 seeds from p6xV2 5

Supplementary Table S1. Editing efficiencies of ttLbCas12a Ultra V2 6

Supplementary Table S2. Editing efficiencies of five Cas12a variants 7

Supplementary Table S3. Editing efficiencies of five Cas12a variants 8

Supplementary Table S4. Analysis of mutations in *TRY* and *CPC* in T-DNA-free T2 plants..........................9

Supplementary Table S5. Analysis of possible off-target mutations 10

Supplementary Table S6. Mutations in the six clade A PP2Cs in T1 transgenic lines 11

Supplementary Table S7. Mutations in the six clade A PP2Cs in T-DNA-free T2 plants 12

Supplementary Table S8. Editing efficiencies of seven LbCas12a variants across four target sites...........13

Supplementary Table S9. Editing efficiencies of one Cas12i3 variant and two Cas12f variants 14

Supplementary Table S10. Primer sequences used in this study...15

Supplementary Table S11. Sequences of target sites for LbCas12a or Cas12i3 16

Supplementary Table S12. Sequences of target sites for Cas12f variants 18

Supplementary Table S13. List of binary vectors used in this study..19

Supplementary Figure S1. Germination delay and growth defects of T1 seeds from p6xV1

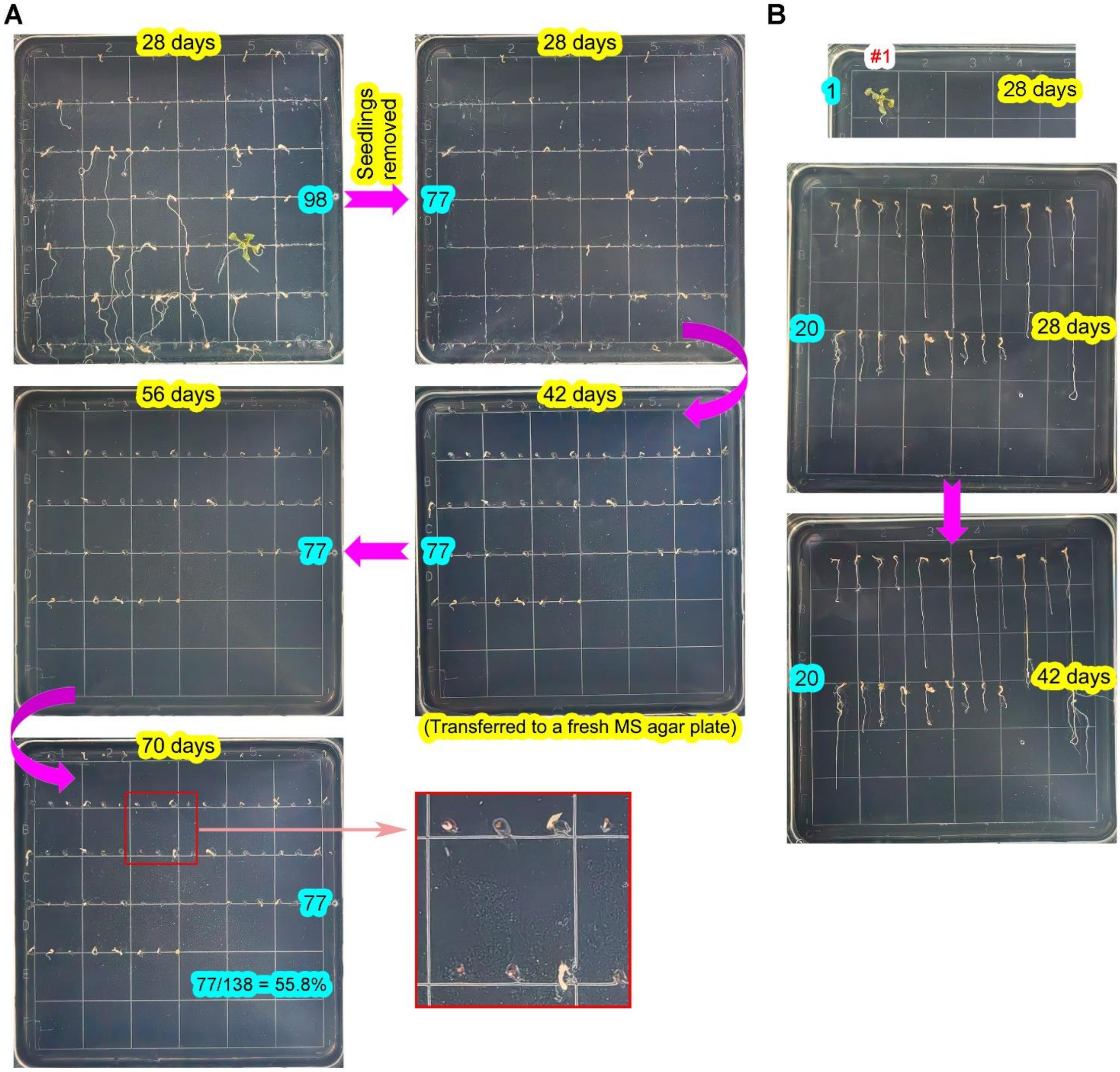

Supplementary Figure S1. Germination delay and growth defects of T1 seeds from p6xV1 after 28, 42, 56, and 70 days of cultivation**. A)** Seedlings were removed to fresh MS plates after 28, 42, 56, and 70 days of cultivation. Photographs were taken before and after the seedlings were removed. No seedlings were available after 42 days of cultivation, and seeds that did not germinate well were moved to a fresh MS plate. The numbers highlighted in blue represent numbers of seeds and seedlings in plates. The side length of the squares at the bottom of plates is 1.4 cm. **B)** Seedlings cultivated for 28 days were removed to fresh MS plates and photographed. The image of the plate with only one seedling is the same as that in Fig. 2C.

Supplementary Figure S2. The optimized LbCas12a variant efficiently generates

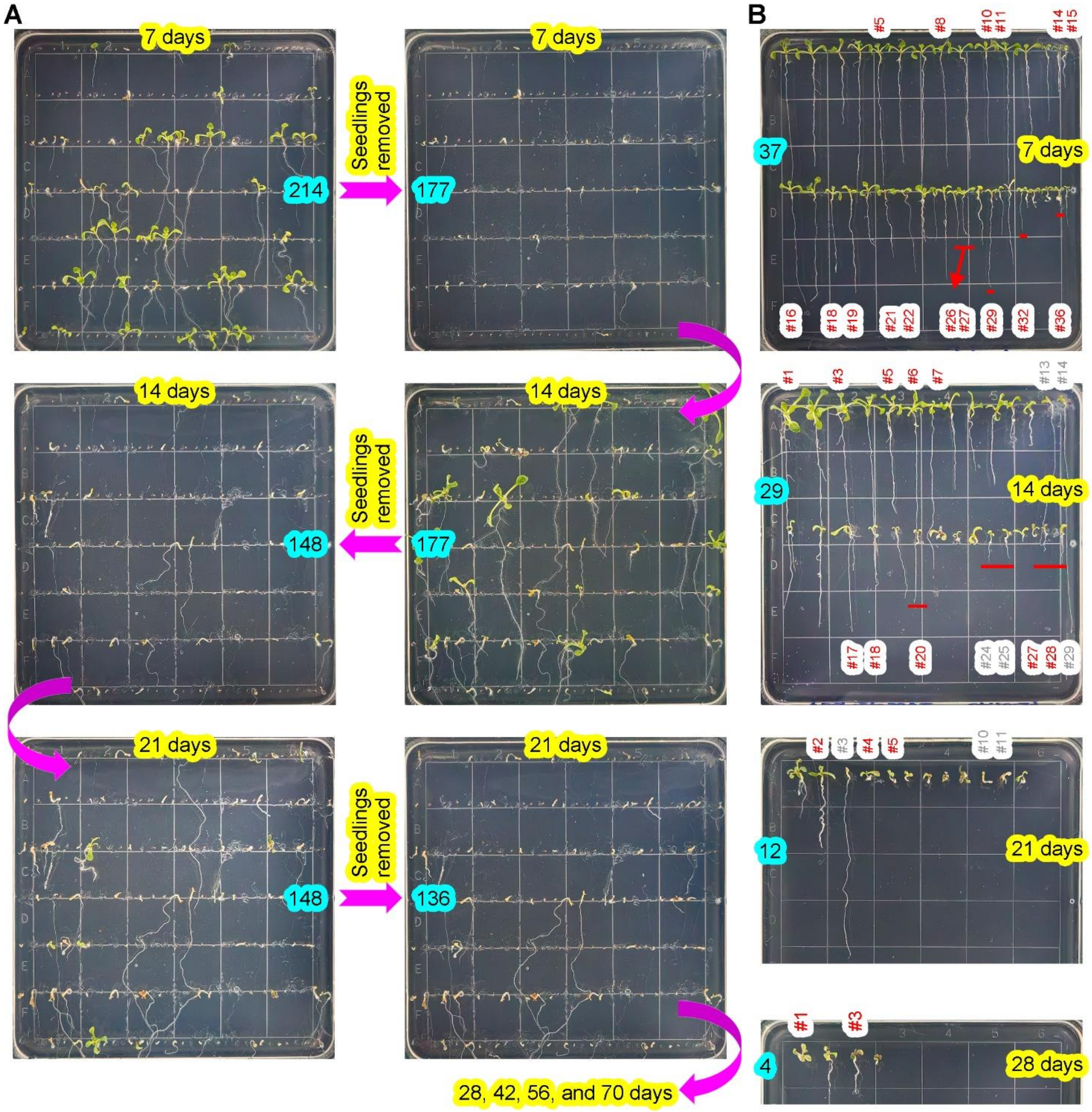

Supplementary Figure S2. The optimized LbCas12a variant efficiently generates homozygous sextuple mutants in clade A PP2C genes. **A)** The majority of T1 seeds from p6xV2 displayed retarded germination and growth defects**.** Seedlings were removed to fresh MS plates every 7 days and pictures were taken before and after their removal. The numbers highlighted in blue represent numbers of seeds and seedlings in plates. The side length of the squares at the bottom of plates is 1.4 cm. **B)** Seedlings were removed to fresh MS plates, photographed, and transplanted into soil after 0–7 days of cultivation on MS agar plates. The genotypes of plants in soil were determined by high-throughput sequencing of PCR amplicons. Line numbers in red indicate homozygous sextuple mutants; line numbers in gray indicate lines that died after transplantation into soil.

Supplementary Figure S3. Germination delay and growth defects of T1 seeds from p6xV2

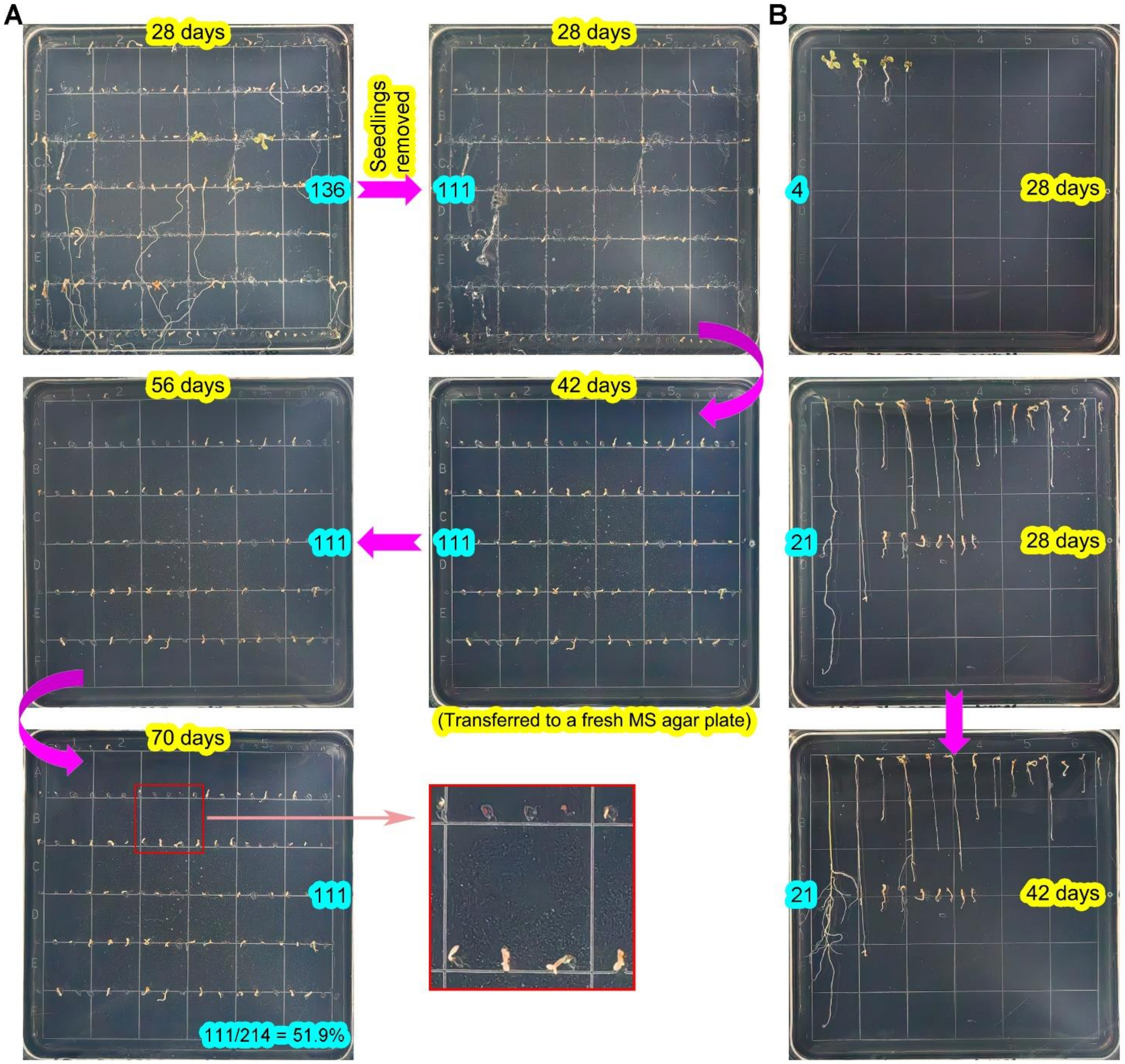

Supplementary Figure S3. Germination delay and growth defects of T1 seeds from p6xV2 after 28, 42, 56, and 70 days of cultivation**. A)** Seedlings were removed to fresh MS plates after 28, 42, 56, and 70 days of cultivation. Photographs were taken before and after the seedlings were removed. No seedlings were available after 42 days of cultivation, and seeds that did not germinate well were transferred to a fresh MS plate. The numbers highlighted in blue represent numbers of seeds and seedlings in plates. The side length of the squares at the bottom of plates is 1.4 cm. **B)** Seedlings after cultivation for 28 days were removed to fresh MS plates and photographed. The image of the plate with four seedlings is the same as that in Supplementary Fig. S2.

Supplementary Table S1. Editing efficiencies of ttLbCas12a Ultra V2

Supplementary Table S1. Editing efficiencies of *ttLbCas12a Ultra V2* across 12 GL1 target sites

Target	Ratio of Ho/Bi	Ratio of Chi	Total	Predicted
#1 (GL1-1)	86.2% (326/378)	2.9% (11/378)	89.2% (337/378)	88%
#2 (GL1-2)	98.9% (275/278)	1.1% (3/278)	100.0% (278/278)	38%
#3 (GL1-3)	99.1% (214/216)	0.0% (0/216)	99.1% (214/216)	64%
#4 (GL1-4)	30.2% (65/215)	47.0% (101/215)	77.2% (166/215)	50%
#5 (GL1-5)	78.1% (175/224)	19.6% (44/224)	97.8% (219/224)	41%
#6 (GL1-6)	95.0% (95/100)	2.0% (2/100)	97.0% (97/100)	23%
#7 (GL1-7)	98.8% (161/163)	0.6% (1/163)	99.4% (162/163)	67%
#8 (GL1-8)	45.9% (61/133)	33.1% (44/133)	78.9% (105/133)	60%
#9 (GL1-9)	44.8% (56/125)	39.2% (49/125)	84.0% (105/125)	45%
#10 (GL1-10)	28.4% (42/148)	32.4% (48/148)	60.8% (90/148)	52%
#11 (GL1-11)	40.6% (54/133)	35.3% (47/133)	75.9% (101/133)	63%
#12 (GL1-12)	20.8% (26/125)	38.4% (48/125)	59.2% (74/125)	66%

Data for the two targets #1 (GL1-1) and #2 (GL1-2) are from the previous report and data for the GL1-1 target are derived from the less efficient ttLbCas12a Ultra variant in combination with the U6-tRNA(Gly) cassette.

Supplementary Table S2. Editing efficiencies of five Cas12a variants

Supplementary Table S2. Editing efficiencies of five Cas12a variants in generating *chli1 chli2* double mutants

crRNAs	Cas12a variant	Strong	Moderate	Weak	Total
1&2-1	ttLbCas12a Ultra V2	85.4% (252/295)	10.2% (30/295)	1.0% (3/295)	96.6% (285/295)
	LbCas12a-RRV	89.1% (57/64)	6.3% (4/64)	0.0% (0/64)	95.3% (61/64)
	LbCas12a-RRVL	94.7% (144/152)	2.6% (4/152)	0.0% (0/152)	97.4% (148/152)
	hyperLbCas12a	67.7% (65/96)	25.0% (24/96)	0.0% (0/96)	92.7% (89/96)
	hyperLbCas12a Ultra	59.8% (98/164)	36.0% (59/164)	0.0% (0/164)	95.7% (157/164)
1&2-2	ttLbCas12a Ultra V2	95.3% (182/191)	1.6% (3/191)	1.0% (2/191)	97.9% (187/191)
	LbCas12a-RRV	98.8% (83/84)	0.0% (0/84)	1.2% (1/84)	100.0% (84/84)
	LbCas12a-RRVL	99.0% (98/99)	0.0% (0/99)	0.0% (0/99)	99.0% (98/99)
	hyperLbCas12a	92.9% (197/212)	4.7% (10/212)	0.9% (2/212)	98.6% (209/212)
	hyperLbCas12a Ultra	93.8% (180/192)	5.2% (10/192)	0.0% (0/192)	99.0% (190/192)
1&2-3	ttLbCas12a Ultra V2	99.3% (300/302)	0.7% (2/302)	0.0% (0/302)	100.0% (302/302)
	LbCas12a-RRV	99.3% (137/138)	0.0% (0/138)	0.0% (0/138)	99.3% (137/138)
	LbCas12a-RRVL	98.9% (93/94)	0.0% (0/94)	0.0% (0/94)	98.9% (93/94)
	hyperLbCas12a	98.2% (108/110)	1.8% (2/110)	0.0% (0/110)	100.0% (110/110)
	hyperLbCas12a Ultra	99.4% (155/156)	0.0% (0/156)	0.0% (0/156)	99.4% (155/156)

Plants with strong phenotypes were regarded as homozygous or biallelic mutants, and those with moderate or weak phenotypes were regarded as chimeric mutants.

Supplementary Table S3. Editing efficiencies of five Cas12a variants

Supplementary Table S3. Editing efficiencies of five Cas12a variants in generating *try cpc* double mutants

crRNAs	Cas12a variant	Strong	Moderate	Weak	Total
T&C	ttLbCas12a Ultra V2	97.8% (132/135)	0.7% (1/135)	0.0% (0/135)	98.5% (133/135)
	LbCas12a-RRV	97.7% (126/129)	0.8% (1/129)	1.6% (2/129)	100.0% (129/129)
	LbCas12a-RRVL	100.0% (136/136)	0.0% (0/136)	0.0% (0/136)	100.0% (136/136)
	hyperLbCas12a	94.4% (119/126)	3.2% (4/126)	0.8% (1/126)	98.4% (124/126)
	hyperLbCas12a Ultra	95.6% (109/114)	2.6% (3/114)	0.9% (1/114)	99.1% (113/114)
C&T	ttLbCas12a Ultra V2	96.1% (148/154)	1.3% (2/154)	1.9% (3/154)	99.4% (153/154)
	LbCas12a-RRV	96.2% (101/105)	1.9% (2/105)	0.0% (0/105)	98.1% (103/105)
	LbCas12a-RRVL	97.8% (134/137)	1.5% (2/137)	0.7% (1/137)	100.0% (137/137)
	hyperLbCas12a	93.9% (138/147)	3.4% (5/147)	0.7% (1/147)	98.0% (144/147)
	hyperLbCas12a Ultra	96.2% (177/184)	1.6% (3/184)	1.1% (2/184)	98.9% (182/184)

Plants with strong phenotypes were regarded as homozygous or biallelic mutants, and those with moderate or weak phenotypes were regarded as chimeric mutants.

Supplementary Table S4. Analysis of mutations in *TRY* and *CPC* in T-DNA-free T2 plants

Supplementary Table S4. Analysis of mutations in *TRY* and *CPC* in T-DNA-free T2 plants

Vectors	T1 line	Ratio of T-DNA-free T2 double mutants
pV2-T&C	#2	100% (55/55)
	#6	100% (33/33)
	#10	100% (45/45)
	#39	100% (40/40)
	#44	100% (31/31)
	#47	100% (24/24)
pV2-C&T	#1	100% (12/12)
	#2	100% (66/66)
	#15	100% (44/44)
	#16	100% (53/53)
	#28	100% (19/19)
	#36	100% (63/63)

Supplementary Table S5. Analysis of possible off-target mutations

Supplementary Table S5. Analysis of off-target mutations

Target or off-target gene	Sequence of target or off-target	No. of mismatches	No. of mutants analyzed	No. of mutants with off-target mutations
TRY	**TTTC**TCATTATCCAATATCTCTCTATC	/	/	/
At2G30432	**TTTC**TCATaATCCAgTATCTCTCTATC	2	36	0
At2G30420	**TTTC**TCATaATCCAgTATCTCTCaATC	3	36	0
At2G30424	**TTTC**TCATaATCCAATATCTtTCTATg	3	36	0

Homozygous or biallelic mutants carrying pV2-T&C were analyzed for off-target mutations. PAMs are bold and underlined, while mismatched bases are in lowercase and red.

Supplementary Table S6. Mutations in the six clade A PP2Cs in T1 transgenic lines

Supplementary Table S6. Mutations in the six clade A PP2Cs in T1 transgenic lines
(See the XLSX spreadsheet)

Supplementary Table S7. Mutations in the six clade A PP2Cs in T-DNA-free T2 plants

Supplementary Table S7. Mutations in the six clade A PP2Cs in T-DNA-free T2 plants

(See the XLSX spreadsheet)

Supplementary Table S8. Editing efficiencies of seven LbCas12a variants across four target sites

Supplementary Table S8. Editing efficiencies of seven LbCas12a variants across four target sites

Target	Cas12a variant	Ratio of Ho/Bi	Ratio of He	Ratio of Chi	Total ratio
ECA3-1	ttLbCas12a Ultra V0	1.1% (1/92)	19.6% (18/92)	53.3% (49/92)	73.9% (68/92)
	ttLbCas12a Ultra V1	69.8% (67/96)	25.0% (24/96)	4.2% (4/96)	99.0% (95/96)
	ttLbCas12a Ultra V2	86.4% (76/88)	11.4% (10/88)	1.1% (1/88)	98.9% (87/88)
	LbCas12a-RRV	98.9% (92/93)	0.0% (0/93)	0.0% (0/93)	98.9% (92/93)
	LbCas12a-RRVL	97.8% (90/92)	1.1% (1/92)	0.0% (0/92)	98.9% (91/92)
	hyperLbCas12a	68.5% (63/92)	19.6% (18/92)	10.9% (10/92)	98.9% (91/92)
	hyperLbCas12a Ultra	81.0% (68/84)	11.9% (10/84)	2.4% (2/84)	95.2% (80/84)
GL2	ttLbCas12a Ultra V0	0.0% (0/137)	1.5% (2/137)	40.9% (56/137)	42.3% (58/137)
	ttLbCas12a Ultra V1	25.0% (24/96)	22.9% (22/96)	49.0% (47/96)	96.9% (93/96)
	ttLbCas12a Ultra V2	21.3% (30/141)	17.0% (24/141)	60.3% (85/141)	98.6% (139/141)
	LbCas12a-RRV	27.1% (26/96)	42.7% (41/96)	29.2% (28/96)	99.0% (95/96)
	LbCas12a-RRVL	62.1% (59/95)	23.2% (22/95)	13.7% (13/95)	98.9% (94/95)
	hyperLbCas12a	0.0% (0/96)	12.5% (12/96)	67.7% (65/96)	80.2% (77/96)
	hyperLbCas12a Ultra	4.3% (4/94)	14.9% (14/94)	60.6% (57/94)	79.8% (75/94)
TT4	ttLbCas12a Ultra V0	8.6% (8/93)	18.3% (17/93)	51.6% (48/93)	78.5% (73/93)
	ttLbCas12a Ultra V1	71.9% (69/96)	16.7% (16/96)	9.4% (9/96)	97.9% (94/96)
	ttLbCas12a Ultra V2	86.2% (81/94)	9.6% (9/94)	4.3% (4/94)	100.0% (94/94)
	LbCas12a-RRV	97.5% (78/80)	2.5% (2/80)	0.0% (0/80)	100.0% (80/80)
	LbCas12a-RRVL	98.9% (86/87)	1.1% (1/87)	0.0% (0/87)	100.0% (87/87)
	hyperLbCas12a	68.5% (61/89)	22.5% (20/89)	7.9% (7/89)	98.9% (88/89)
	hyperLbCas12a Ultra	90.4% (85/94)	7.4% (7/94)	1.1% (1/94)	98.9% (93/94)
GL1-2	ttLbCas12a Ultra V0	84.9% (242/285)	/	10.5% (30/285)	95.4% (272/285)
	ttLbCas12a Ultra V1	97.2% (209/215)	/	0.9% (2/215)	98.1% (211/215)
	ttLbCas12a Ultra V2	98.9% (275/278)	/	1.1% (3/278)	100.0% (278/278)
	LbCas12a-RRV	100.0% (187/187)	/	0.0% (0/187)	100.0% (187/187)
	LbCas12a-RRVL	98.6% (145/147)	/	1.4% (2/147)	100.0% (147/147)
	hyperLbCas12a	92.4% (159/172)	/	1.7% (3/172)	94.2% (162/172)
	hyperLbCas12a Ultra	97.0% (192/198)	/	1.0% (2/198)	98.0% (194/198)

Supplementary Table S9. Editing efficiencies of one Cas12i3 variant and two Cas12f variants

Supplementary Table S9. Editing efficiencies of one Cas12i3 variant and two Cas12f variants across six target sites

Target	Cas variant	Ho/Bi	He	Chi	Total
ECA3-1	Cas12i3V1	0.0% (0/96)	1.0% (1/96)	36.5% (35/96)	37.5% (36/96)
	Cas12i3V2	0.0% (0/96)	0.0% (0/96)	22.9% (22/96)	22.9% (22/96)
	AsCas12f-YHAM	0.0% (0/94)	0.0% (0/94)	1.1% (1/94)	1.1% (1/94)
	AsCas12f-HKRA	0.0% (0/96)	0.0% (0/96)	0.0% (0/96)	0.0% (0/96)
ECA3-2	Cas12i3V1	0.0% (0/96)	15.6% (15/96)	42.7% (41/96)	58.3% (56/96)
	Cas12i3V2	0.0% (0/94)	4.3% (4/94)	30.9% (29/94)	35.1% (33/94)
	AsCas12f-YHAM	0.0% (0/96)	0.0% (0/96)	0.0% (0/96)	0.0% (0/96)
	AsCas12f-HKRA	0.0% (0/96)	0.0% (0/96)	0.0% (0/96)	0.0% (0/96)
GL2	Cas12i3V1	55.2% (53/96)	42.7% (41/96)	2.1% (2/96)	100.0% (96/96)
	Cas12i3V2	36.5% (35/96)	51.0% (49/96)	12.5% (12/96)	100.0% (96/96)
	AsCas12f-YHAM	0.0% (0/96)	1.0% (1/96)	12.5% (12/96)	13.5% (13/96)
	AsCas12f-HKRA	0.0% (0/96)	0.0% (0/96)	0.0% (0/96)	0.0% (0/96)
TT4	Cas12i3V1	0.0% (0/94)	2.1% (2/94)	21.3% (20/94)	23.4% (22/94)
	Cas12i3V2	0.0% (0/96)	1.0% (1/96)	32.3% (31/96)	33.3% (32/96)
	AsCas12f-YHAM	0.0% (0/96)	0.0% (0/96)	0.0% (0/96)	0.0% (0/96)
	AsCas12f-HKRA	0.0% (0/96)	0.0% (0/96)	0.0% (0/96)	0.0% (0/96)
GL1-1	Cas12i3V1	73.0% (46/63)	/	20.6% (13/63)	93.7% (59/63)
	Cas12i3V2	50.8% (92/181)	/	36.5% (66/181)	87.3% (158/181)
	AsCas12f-YHAM	0.0% (0/103)	/	0.0% (0/103)	0.0% (0/103)
	AsCas12f-HKRA	0.0% (0/98)	/	0.0% (0/98)	0.0% (0/98)
GL1-2	Cas12i3V1	26.6% (29/109)	/	37.6% (41/109)	64.2% (70/109)
	Cas12i3V2	12.3% (14/114)	/	23.7% (27/114)	36.0% (41/114)
	AsCas12f-YHAM	0.0% (0/215)	/	0.0% (0/215)	0.0% (0/215)
	AsCas12f-HKRA	0.0% (0/131)	/	0.0% (0/131)	0.0% (0/131)

Cas12i3V1 and Cas12i3V2 are identical Cas12i3 variants but differ in codon usage. Ho/Bi, homozygous or biallelic. He, heterozygous. Chi, chimeric.

Supplementary Table S10. Primer sequences used in this study

Supplementary Table S10. Primer sequences used in this study
(See the XLSX spreadsheet)

Supplementary Table S11. Sequences of target sites for LbCas12a or Cas12i3

Supplementary Table S11. Sequences of target sites for LbCas12a or Cas12i3

Gene	Target	PAM	Guide (23-nt for all but ECA3-1/2)
AtECA3	ECA3-1	TTTC	CTAGCTTATCGTGATAGATCTTCC
	ECA3-2	TTTA	AGCAATTATACTATCAGATATCCG
AtGL2	GL2	TTTG	TATGTCAATGGCCGTCGACATGT
AtTT4	TT4	TTTA	CTATTCACAGGCGACAAGTCGAC
AtCHLI1 *AtCHLI2*	CHLI1&2-1	TTTA	CCAGTTCCTCTATCTCCCATTAT
	CHLI1&2-2	TTTG	GGTTACTATCGAAACGAGCTCTC
	CHLI1&2-3	TTTA	TGTTGATGAAGTTAATCTCTTGG
AtTRY *AtCPC*	T&C	TTTC	TCATTATCCAATATCTCTCTATC
		TTTC	ATAAGCCAATATCTCTCTATCTC
	C&T	TTTC	ATAAGCCAATATCTCTCTATCTC
		TTTC	TCATTATCCAATATCTCTCTATC
AtGL1	GL1-1	TTTC	ACTGAACAAGAAGAAGACCTCAT
	GL1-2	TTTG	ATAGCTAAAAGAGTACCGGGAAG
	GL1-3	TTTA	TGGACAGTTGAAGAAGACAACAT
	GL1-4	TTTC	TGACGATGCGGTTCCATTGGCCA
	GL1-5	TTTG	AGCCCTAATGTGAACAAAGGCAA
	GL1-6	TTTG	TTCACATTAGGGCTCAAATAATT
	GL1-7	TTTG	CTGAGATGAGTGTTCCAGTAGTT
	GL1-8	TTTG	ACGGCGGAGGAGTAATCTCCGAC
	GL1-9	TTTC	TTGTTGATGATGACAAGAAGAAG
	GL1-10	TTTA	ACGGCGTCGTATCAGCTTCGTAC
	GL1-11	TTTA	TCCTCGTACGAAGCTGATACGAC
	GL1-12	TTTG	GTTTATCCTCGTACGAAGCTGAT
AtAHG1	AHG1	*TTTC*	AAGAGGATGGATGAGATGGCAAC
AtAHG3	AHG3	*TTTC*	ATACTCCGAGTAGCACCATTAAC
AtHAI1	HAI1	*TTTC*	CCAGTCAGCATCAGCTTCAAACT
AtHAI2	HAI2	*TTTG*	AAGCTCACACCTACAATTAGCAC
AtHAI3	HAI3	*TTTG*	*CAGTCACGACGGAATCACCCCAC*

AtHAB2	HAB2	*TTTG*	AGACTATTATATGCGATGAACAA

Supplementary Table S12. Sequences of target sites for Cas12f variants

Supplementary Table S12. Sequences of target sites for Cas12f variants

**Gene**	**Target**	**PAM**	**Guide (20-nt)**
AtECA3	ECA3-1	TTTC	CTAGCTTATCGTGATAGATC
	ECA3-2	TTTA	AGCAATTATACTATCAGATA
AtGL2	GL2	TTTG	TATGTCAATGGCCGTCGACA
AtTT4	TT4	TTTA	CTATTCACAGGCGACAAGTC
AtGL1	GL1-1	TTTC	ACTGAACAAGAAGAAGACCT
	GL1-2	TTTG	ATAGCTAAAAGAGTACCGGG

Supplementary Table S13. List of binary vectors used in this study

Supplementary Table S13. List of binary vectors used in this study
(See the XLSX spreadsheet)
