# Supplementary material for: Never-germinating Arabidopsis seeds with LbCas12a-induced mutations in 6 clade A type 2C protein phosphatase genes
Source: Plant Physiol. 2025 Jul 17;198(3):kiaf315. doi: 10.1093/plphys/kiaf315 (PMC12301882; doi:10.1093/plphys/kiaf315)
Supplement: kiaf315_Supplementary_Data [file kiaf315_supplementary_data.zip › Xin et al. 2025 PP R2 Supplementary Sequences.pdf]

# Supplementary Sequences

## Never-Germinating Arabidopsis Seeds with LbCas12a-Induced Mutations in Six Clade A Type 2C Protein Phosphatase Genes

Cuiping **Xin**,[1] Yu **Lu**,[1] Syeda Leeda **Gul**,[1] Wei **Sun**,[1] Zhenghong **Cao**,[1] Xiangchao **Kong**,[1] Kexin **Fan**,[1] Siyun **Li**,[1] Xiaohan **Liu**,[1] Xue-Chen **Wang**,[1] Qi-Jun **Chen**[1,2,*]

[1]State Key Laboratory of Plant Environmental Resilience, College of Biological Sciences, China Agricultural University, Beijing 100193, China

[2]Center for Crop Functional Genomics and Molecular Breeding, China Agricultural University, Beijing 100193, China

[*] Corresponding author: qjchen@cau.edu.cn

## Table of contents

Supplementary Sequences S1. Sequences of Cas12 variants and crRNA/sgRNA cassettes .................. 3

Sequence of ttLbCas12a Ultra (ttLbUV0) .................. 3

Sequence of ttLbCas12a Ultra V1 (ttLbUV1) .................. 4

Sequence of ttLbCas12a Ultra V2 (ttLbUV2) .................. 4

Sequence of LbCas12a-RRV .................. 6

Sequence of LbCas12a-RRVL .................. 6

Sequence of hyperCas12a .................. 6

Sequence of hyperCas12a Ultra .................. 7

Sequence of Cas12i3V1 .................. 7

Sequence of Cas12i3V2 .................. 9

Sequence of AsCas12f-YHAM .................. 10

Sequence of AsCas12f-HKRA .................. 11

Cloning cassette for assembly of LbCas12a crRNA .................. 12

Final cassette for expression of 1×crRNA for LbCas12a .................. 12

Final cassette for expression of 2×crRNAs for LbCas12a .................. 12

Final cassette for expression of 3×crRNAs for LbCas12a .................. 14

Cloning cassette in pAGC-Lb12-P1Bb .................. 15

Cloned sequence in pAGC-S1–S4 .................. 15

Final cassette for expression of 4×crRNAs for LbCas12a .................. 16

Cloned sequence in pAGC-S1–S6 .................. 17

Final cassette for expression of 6×crRNAs for LbCas12a .................. 17

Cloning cassette for assembly of Cas12i3 crRNA .................. 19

Final cassette for expression of a Cas12i3 crRNA .................. 19

Cloning cassette for assembly of AsCas12f sgRNA .................. 20

Final cassette for expression of an AsCas12f sgRNA .................. 20

## Supplementary Sequences S1. Sequences of Cas12 variants and crRNA/sgRNA cassettes

### Sequence of ttLbCas12a Ultra (ttLbUV0)

NLS$^{SV40}$-ttLbUV0-bpNLS$^{Npl}$-linker-1×HA (Npl, the *Xenopus Laevis* protein nucleoplasmin)

ATGGCCCCGAAGAAGAAGCGCAAAGTGGGCATCCACGGCGTGCCAGCCGCCTCCAAGCTCGAGAA
GTTCACGAACTGCTACTCCCTCAGCAAGACCCTGAGGTTCAAGGCCATCCCGGTCGGCAAGACCCA
GGAGAACATCGACAACAAGCGCCTCCTGGTCGAGGACGAGAAGAGGGCGGAGGACTACAAGGGC
GTGAAGAAGCTCCTGGACCGCTACTACCTCTCCTTCATCAACGACGTGCTGCACAGCATCAAGCTCA
AGAACCTGAACAACTACATCTCCCTGTTCCGCAAGAAGACGAGGACCGAGAAGGAGAACAAGGAGC
TGGAGAACCTGGAGATCAACCTCAGGAAGGAGATCGCCAAGGCGTTCAAGGGCAACGAGGGCTAC
AAGAGCCTGTTCAAGAAGGACATCATCGAGACGATCCTCCCCGAGTTCCTGGACGACAAGGACGAG
ATCGCCCTCGTCAACTCCTTCAACGGCTTCACCACGGCGTTCACCGGCTTCTTCCGCAACCGCGAG
AACATGTTCAGCGAGGAGGCCAAGTCCACGAGCATCGCGTTCCGCTGCATCAACGAGAACCTGACC
AGGTACATCTCCAACATGGACATCTTCGAGAAGGTCGACGCCATCTTCGACAAGCACGAGGTGCAG
GAGATCAAGGAGAAGATCCTCAACAGCGACTACGACGTCGAGGACTTCTTCGAGGGCGAGTTCTTC
AACTTCGTCCTGACGCAGGAGGGCATCGACGTGTACAACGCGATCATCGGTGGCTTCGTGACCGAG
TCCGGCGAGAAGATCAAGGGCCTCAACGAGTACATCAACCTGTACAACCAGAAGACCAAGCAGAAG
CTCCCGAAGTTCAAGCCCCTCTACAAGCAGGTGCTGTCCGACCGCGAGTCCCTGAGCTTCTACGGC
GAGGGCTACACGAGCGACGAGGAGGTCCTCGAGGTGTTCAGGAACACCCTGAACAAGAACAGCGA
GATCTTCTCCAGCATCAAGAAGCTCGAGAAGCTGTTCAAGAACTTCGACGAGTACTCCAGCGCCGG
CATCTTCGTCAAGAACGGCCCGGCGATCTCCACGATCAGCAAGGACATCTTCGGCGAGTGGAACGT
GATCCGCGACAAGTGGAACGCCGAGTACGACGACATCCACCTCAAGAAGAAGGCGGTGGTCACCG
AGAAGTACGAGGACGACCGCAGGAAGTCCTTCAAGAAGATCGGCTCCTTCAGCCTCGAGCAGCTG
CAGGAGTACGCCGACGCGGACCTCAGCGTGGTCGAGAAGCTGAAGGAGATCATCATCCAGAAGGT
CGACGAGATCTACAAGGTGTACGGCTCCAGCGAGAAGCTGTTCGACGCCGACTTCGTCCTCGAGAA
GTCCCTGAAGAAGAACGACGCCGTGGTCGCGATCATGAAGGACCTCCTGGACTCCGTGAAGAGCTT
CGAGAATTACATCAAGGCGTTCTTCGGCGAGGGCAAGGAGACGAACCGCGACGAGTCCTTCTACGG
CGACTTCGTCCTCGCCTACGACATCCTCCTGAAGGTGGACCACATCTACGACGCGATCAGGAACTAC
GTGACCCAGAAGCCGTACAGCAAGGACAAGTTCAAGCTGTACTTCCAGAACCCCCAGTTCATGGGC
GGCTGGGACAAGGACAAGGAGACGGACTACCGCGCCACCATCCTCCGCTACGGCAGCAAGTACTA
CCTGGCCATCATGGACAAGAAGTACGCGAAGTGCCTCCAGAAGATCGACAAGGACGACGTCAACGG
CAACTACGAGAAGATCAACTACAAGCTCCTGCCGGGCCCCAACAAGATGCTGCCGAAGGTGTTCTT
CTCCAAGAAGTGGATGGCCTACTACAACCCCAGCGAGGACATCCAGAAGATCTACAAGAACGGCAC
GTTCAAGAAGGGCGACATGTTCAACCTCAACGACTGCCACAAGCTGATCGACTTCTTCAAGGACTCC
ATCAGCCGCTACCCGAAGTGGTCCAACGCCTACGACTTCAACTTCAGCGAGACGGAGAAGTACAAG
GACATCGCGGGGTTCTACAGGGAGGTCGAGGAGCAGGGCTACAAGGTGTCCTTCGAGTCCGCCAG
CAAGAAGGAGGTCGACAAGCTCGTGGAGGAGGGCAAGCTGTACATGTTCCAGATCTACAACAAGGA
CTTCTCCGACAAGAGCCACGGCACGCCCAACCTCCACACCATGTACTTCAAGCTCCTGTTCGACGA
GAACAACCACGGCCAGATCAGGCTCTCCGGCGGCGCCGAGCTGTTCATGCGCAGGGCGAGCCTCA
AGAAGGAGGAGCTGGTGGTCCACCCCGCCAACAGCCCAATCGCGAACAAGAACCCGGACAACCCC
AAGAAGACCACGACCCTCTCCTACGACGTGTACAAGGACAAGCGCTTCAGCGAGGACCAGTACCTG
CTGCACATCCCGATCGCCATCAACAAGTGCCCCAAGAACATCTTCAAGATCAACACCGAGGTCAGG
GTGCTCCTGAAGCACGACGACAACCCCTACGTGATCGGCATCGACAGGGGCGAGAGGAACCTCCT

GTACATCGTGGTCGTGGACGGCAAGGGCAACATCGTGGAGCAGTACTCCCTGAACGAGATCATCAA
CAACTTCAACGGCATCCGCATCAAGACGGACTACCACAGCCTCCTGGACAAGAAGGAGAAGGAGCG
CTTCGAGGCCAGGCAGAACTGGACCTCCATCGAGAACATCAAGGAGCTGAAGGCGGGCTACATCA
GCCAGGTCGTGCACAAGATCTGCGAGCTGGTCGAGAAGTACGACGCCGTGATCGCGCTCGAGGAC
CTGAACTCCGGCTTCAAGAACAGCAGGGTCAAGGTGGAGAAGCAGGTCTACCAGAAGTTCGAGAA
GATGCTCATCGACAAGCTGAACTACATGGTGGACAAGAAGTCCAACCCCTGCGCTACGGGCGGCGC
GCTCAAGGGCTACCAGATCACCAACAAGTTCGAGAGCTTCAAGTCCATGAGCACTCAGAACGGCTT
CATCTTCTACATCCCGGCCTGGCTGACGTCCAAGATCGACCCCAGCACCGGCTTCGTCAACCTCCT
GAAGACGAAGTACACCTCCATCGCGGACAGCAAGAAGTTCATCTCCAGCTTCGACCGCATCATGTAT
GTGCCGGAGGAGGACCTCTTCGAGTTCGCCCTGGACTACAAGAACTTCTCCAGGACGGACGCGGA
CTACATCAAGAAGTGGAAGCTCTACAGCTACGGCAACCGCATCAGGATCTTCCGCAACCCCAAGAAG
AACAACGTCTTCGACTGGGAGGAGGTGTGCCTCACCTCCGCCTACAAGGAGCTGTTCAACAAGTAC
GGCATCAACTACCAGCAGGGCGACATCAGGGCGCTCCTGTGCGAGCAGAGCGACAAGGCGTTCTA
CTCCAGCTTCATGGCGCTCATGTCCCTCATGCTGCAGATGCGCAACAGCATCACGGGCAGGACCGA
CGTCGACTTCCTGATCTCCCCGGTGAAGAACAGCGACGGCATCTTCTACGACTCCCGCAACTACGA
GGCCCAGGAGAACGCGATCCTGCCAAAGAACGCGGACGCCAACGGCGCCTACAACATCGCGAGGA
AGGTGCTGTGGGCCATCGGCCAGTTCAAGAAGGCGGAGGACGAGAAGCTCGACAAGGTCAAGATC
GCCATCTCCAACAAGGAGTGGCTGGAGTACGCGCAGACGAGCGTGAAGCACAAGCGCCCGGCCG
CGACCAAGAAGGCGGGCCAGGCCAAGAAGAAGAAGGGATCCTACCCTTACGACGTGCCAGACTAC
GCTTGA

Codons in red indicate the D156R and E795L mutations.

## Sequence of ttLbCas12a Ultra V1 (ttLbUV1)

bpNLS$^{SV40}$-ttLbUV1-linker- bpNLS$^{SV40}$-linker- NLS$^{c-Myc}$

ATGAAGCGCACCGCCGACGGCAGCGAGTTCGAGAGCCCTAAGAAGAAGCGCAAGGTCAGCAAGCT
CGAGAAGTTCACGAACTGCTACTCCCTCAGCAAGACCCTGAGGTTCAAGGCCATCCCGGTCGGCAA
//CATCGCGAGGAAGGTGCTGTGGGCCATCGGCCAGTTCAAGAAGGCGGAGGACGAGAAGCTCGAC
AAGGTCAAGATCGCCATCTCCAACAAGGAGTGGCTGGAGTACGCGCAGACGAGCGTGAAGCACTCT
GGCGGGTCTAAGCGCACTGCTGATGGCTCTGAGTTCGAGTCACCTAAGAAGAAGCGCAAGGTCGG
CTCTGGCCCTGCTGCTAAGCGCGTCAAGCTCGACTGA

Codons in red indicate the D156R and E795L mutations. "//" means that the sequence from ttLbCas12a Ultra is omitted for brevity.

## Sequence of ttLbCas12a Ultra V2 (ttLbUV2)

bpNLS$^{SV40}$-ttLbUV2-linker- bpNLS$^{SV40}$-linker- NLS$^{c-Myc}$

ATGAAGCGTACCGCTGATGGCAGCGAGTTCGAGAGCCCTAAGAAGAAGCGTAAGGTGTCAAAGCTC
GAGAAGTTCACCAACTGCTACTCCCTGAGCAAGACCCTGAGGTTCAAGGCCATCCCAGTCGGCAAG
ACCCAGGAGAACATCGACAACAAGAGGCTGCTCGTGGAGGACGAGAAGCGTGCCGAGGACTACAA
GGGCGTGAAGAAGCTGCTCGACAGGTACTACCTGTCATTCATCAACGACGTCCTGCACTCAATCAAG
CTGAAGAACCTGAACAACTACATCTCACTGTTCCGTAAGAAGACCAGGACCGAGAAGGAGAACAAG
GAGCTGGAGAACCTGGAGATCAACCTGCGTAAGGAGATCGCCAAGGCTTTCAAGGGCAACGAGGG
CTACAAGTCACTGTTCAAGAAGGACATCATCGAGACAATCCTGCCAGAGTTCCTCGACGACAAGGAC
GAGATCGCCCTGGTCAACTCCTTCAACGGCTTCACCACCGCCTTCACTGGCTTCTTCAGGAACAGA
GAGAACATGTTCTCTGAGGAGGCCAAGTCCACCTCTATCGCCTTCAGGTGCATCAACGAGAACCTCA

CCCGTTACATCTCCAACATGGACATCTTCGAGAAGGTGGACGCCATCTTCGACAAGCACGAGGTGC
AGGAGATCAAGGAGAAGATCCTCAACTCCGACTACGACGTGGAGGACTTCTTCGAGGGTGAGTTCT
TCAACTTCGTGCTCACTCAGGAGGGTATCGACGTGTACAACGCTATCATCGGTGGCTTCGTGACTGA
GTCCGGTGAGAAGATCAAGGGCCTGAACGAGTACATCAACCTGTACAACCAGAAGACCAAGCAGAA
GCTGCCAAAGTTCAAGCCTCTGTACAAGCAGGTCCTGTCAGACAGAGAGTCACTGTCATTCTACGG
CGAGGGCTACACTTCCGACGAGGAGGTCCTGGAGGTGTTCAGGAACACCCTCAACAAGAACTCCG
AGATCTTCTCTAGCATCAAGAAGCTCGAGAAGCTGTTCAAGAACTTCGACGAGTACTCTAGCGCTGG
CATCTTCGTGAAGAACGGACCTGCCATCTCCACCATCTCCAAGGACATCTTCGGCGAGTGGAACGT
GATCAGAGACAAGTGGAACGCCGAGTACGACGATATTCACCTCAAGAAGAAGGCCGTCGTCACCGA
GAAGTACGAGGACGACAGACGTAAGAGCTTCAAGAAGATCGGCTCCTTCAGCCTGGAGCAGCTGCA
GGAGTACGCTGACGCTGACCTGAGCGTCGTCGAGAAGCTGAAGGAGATCATCATCCAGAAGGTCGA
TGAGATCTACAAGGTCTACGGCTCTAGCGAGAAGCTGTTCGACGCTGACTTCGTGCTGGAGAAGTC
ACTGAAGAAGAACGACGCTGTCGTCGCTATCATGAAGGACCTGCTCGACAGCGTGAAGTCATTCGA
GAACTACATCAAGGCTTTCTTCGGCGAGGGCAAGGAGACTAACAGAGACGAGTCATTCTACGGCGA
CTTCGTCCTCGCTTACGACATCCTGCTCAAGGTGGACCACATCTACGACGCCATCAGAAACTACGTG
ACACAGAAGCCCTACAGCAAGGACAAGTTCAAGCTCTACTTCCAGAACCCACAGTTCATGGGCGGC
TGGGACAAGGACAAGGAGACTGACTACAGGGCCACCATCCTGAGGTACGGTTCAAAGTACTACCTG
GCCATCATGGACAAGAAGTACGCCAAGTGCCTCCAGAAGATCGACAAGGACGACGTCAACGGCAAC
TACGAGAAGATCAACTACAAGCTGCTGCCCGGTCCCAACAAGATGCTCCCAAAGGTGTTCTTCAGCA
AGAAGTGGATGGCCTACTACAACCCAAGCGAGGACATCCAGAAGATCTACAAGAACGGCACCTTCAA
GAAGGGCGACATGTTCAACCTGAACGACTGCCACAAGCTGATCGACTTCTTCAAGGACTCAATCAG
CAGATACCCAAAGTGGTCTAACGCCTACGACTTCAACTTCTCCGAGACTGAGAAGTACAAGGACATC
GCCGGCTTCTACCGTGAGGTGGAGGAGCAGGGCTACAAGGTGTCATTCGAGTCCGCCAGCAAGAA
GGAGGTGGACAAGCTCGTGGAGGAGGGCAAGCTCTACATGTTCCAGATCTACAACAAGGACTTCAG
CGACAAGAGCCACGGCACACCTAACCTCCACACTATGTACTTCAAGCTGCTGTTCGACGAGAACAAC
CACGGCCAGATCAGGCTGTCAGGTGGTGCTGAGCTGTTCATGCGTAGAGCCAGCCTCAAGAAGGA
GGAGCTGGTCGTGCACCCTGCTAACTCACCAATCGCCAACAAGAACCCTGACAACCCTAAGAAGAC
TACCACACTGAGCTACGACGTGTACAAGGACAAGAGGTTCTCCGAGGACCAGTACCTGCTGCACAT
CCCTATCGCTATCAACAAGTGCCCAAAGAACATCTTCAAGATCAACACAGAGGTGAGAGTGCTGCTC
AAGCACGACGACAACCCTTACGTCATCGGCATCGACAGAGGCGAGAGGAACCTGCTGTACATCGTC
GTCGTGGACGGCAAGGGCAACATCGTCGAGCAGTACTCCCTGAACGAGATCATCAACAACTTCAAC
GGCATCAGAATCAAGACCGACTACCACTCCCTGCTCGACAAGAAGGAGAAGGAGCGTTTCGAGGCT
AGACAGAACTGGACATCCATCGAGAACATCAAGGAGCTGAAGGCTGGTTACATCTCTCAGGTCGTG
CACAAGATCTGCGAGCTGGTGGAGAAGTACGACGCTGTGATCGCTCTGGAGGACCTCAACTCCGG
CTTCAAGAACTCCAGAGTGAAGGTGGAGAAGCAGGTCTACCAGAAGTTCGAGAAGATGCTGATCGA
CAAGCTCAACTACATGGTCGATAAGAAGTCCAACCCATGCGCCACTGGTGGTGCTCTCAAGGGTTAC
CAGATCACAAACAAGTTCGAGTCCTTCAAGTCCATGTCCACTCAGAACGGCTTCATCTTCTACATCCC
TGCTTGGCTGACCTCTAAGATCGATCCCTCAACCGGTTTCGTCAACCTGCTCAAGACCAAGTACACC
TCCATCGCCGACTCCAAGAAGTTCATCTCTAGCTTCGACAGAATCATGTATGTCCCAGAGGAGGACC
TGTTCGAGTTCGCCCTCGACTACAAGAACTTCTCCAGAACCGACGCCGACTACATCAAGAAGTGGAA
GCTCTACTCCTACGGCAACAGAATCAGGATCTTCAGAAACCCTAAGAAGAACAACGTCTTCGACTGG
GAGGAGGTGTGCCTGACCAGCGCCTACAAGGAGCTGTTCAACAAGTACGGCATCAACTACCAGCAG
GGTGACATCAGAGCCCTGCTGTGCGAGCAGTCTGACAAGGCTTTCTACTCTAGCTTCATGGCTCTGA
TGAGCCTGATGCTCCAGATGAGAAACTCAATCACAGGCAGAACCGACGTGGACTTCCTGATCTCAC

CCGTGAAGAACTCTGACGGCATCTTCTACGACAGCAGGAACTACGAGGCTCAGGAGAACGCTATCC
TGCCAAAGAACGCTGATGCTAACGGTGCTTACAACATCGCTAGGAAGGTCCTGTGGGCTATCGGCC
AGTTCAAGAAGGCTGAGGATGAGAAGCTGGATAAGGTCAAGATCGCTATCAGCAACAAGGAGTGGC
TGGAGTACGCTCAGACATCTGTGAAGCACTCTGGTGGATCTAAGAGGACTGCCGACGGATCTGAGT
TCGAGTCACCCAAGAAGAAGCGTAAGGTCGGATCTGGACCTGCTGCTAAGAGAGTCAAGCTCGACT
GA

Codons in red indicate the D156R and E795L mutations.

## Sequence of LbCas12a-RRV

bpNLS$^{SV40}$-LbCas12a-RRV-linker- bpNLS$^{SV40}$-linker- NLS$^{c\text{-}Myc}$

ATGAAGCGTACCGCTGATGGCAGCGAGTTCGAGAGCCCTAAGAAGAAGCGTAAGGTGTCAAAGCTC
GAGAAGTTCACCAACTGCTACTCCCTGAGCAAGACCCTGAGGTTCAAGGCCATCCCAGTCGGCAAG
ACCCAGGAGAACATCGACAACAAGAGGCTGCTCGTGGAGGACGAGAAGCGTGCCGAGGACTACAA
GGGCGTGAAGAAGCTGCTCGACAGGTACTACCTGTCATTCATCAACGACGTCCTGCACTCAATCAAG
CTGAAGAACCTGAACAACTACATCTCACTGTTCCGTAAGAAGACCAGGACCGAGAAGGAGAACAAG
GAGCTGGAGAACCTGGAGATCAACCTGCGTAAGGAGATCGCCAAGGCTTTCAAGGGCAACGAGGG
CTACAAGTCACTGTTCAAGAAGGACATCATCGAGACAATCCTGCCAGAGTTCCTCGACGACAAGGAC
GAGATCGCCCTGGTCAACTCCTTCAACAGGTTCACCACCGCCTTCACTGGCTTCTTCAGGAACAGA
GAGAACATGTTCTCTGAGGAGGCCAAGTCCACCTCTATCGCCTTCAGGTGCATCAACGAGAACCTCA
CCGTGTACATCTCCAACATGGACATCTTCGAGAAGGTGGACGCCATCTTCGACAAGCACGAGGTGC//
TACCACACTGAGCTACGACGTGTACAAGGACAAGAGGTTCTCCGAGGACCAGTACGAG//

Codons in red indicate the D156R, G146R, and R182V mutations. “//” means that the sequence from ttLbCas12a Ultra V2 is omitted for brevity.

## Sequence of LbCas12a-RRVL

bpNLS$^{SV40}$-LbCas12a-RRVL-linker- bpNLS$^{SV40}$-linker- NLS$^{c\text{-}Myc}$

ATGAAGCGTACCGCTGATGGCAGCGAGTTCGAGAGCCCTAAGAAGAAGCGTAAGGTGTCAAAGCTC
GAGAAGTTCACCAACTGCTACTCCCTGAGCAAGACCCTGAGGTTCAAGGCCATCCCAGTCGGCAAG
ACCCAGGAGAACATCGACAACAAGAGGCTGCTCGTGGAGGACGAGAAGCGTGCCGAGGACTACAA
GGGCGTGAAGAAGCTGCTCGACAGGTACTACCTGTCATTCATCAACGACGTCCTGCACTCAATCAAG
CTGAAGAACCTGAACAACTACATCTCACTGTTCCGTAAGAAGACCAGGACCGAGAAGGAGAACAAG
GAGCTGGAGAACCTGGAGATCAACCTGCGTAAGGAGATCGCCAAGGCTTTCAAGGGCAACGAGGG
CTACAAGTCACTGTTCAAGAAGGACATCATCGAGACAATCCTGCCAGAGTTCCTCGACGACAAGGAC
GAGATCGCCCTGGTCAACTCCTTCAACAGGTTCACCACCGCCTTCACTGGCTTCTTCAGGAACAGA
GAGAACATGTTCTCTGAGGAGGCCAAGTCCACCTCTATCGCCTTCAGGTGCATCAACGAGAACCTCA
CCGTGTACATCTCCAACATGGACATCTTCGAGAAGGTGGACGCCATCTTCGACAAGCACGAGGTGC//
TACCACACTGAGCTACGACGTGTACAAGGACAAGAGGTTCTCCGAGGACCAGTACCTG//

Codons in red indicate the D156R, G146R, R182V and E795L mutations. “//” means the sequence from ttLbCas12a Ultra V2 is omitted for brevity.

## Sequence of hyperCas12a

bpNLS$^{SV40}$-hyperCas12a-linker- bpNLS$^{SV40}$-linker- NLS$^{c\text{-}Myc}$

ATGAAGCGTACCGCTGATGGCAGCGAGTTCGAGAGCCCTAAGAAGAAGCGTAAGGTGTCAAAGCTC
GAGAAGTTCACCAACTGCTACTCCCTGAGCAAGACCCTGAGGTTCAAGGCCATCCCAGTCGGCAAG

ACCCAGGAGAACATCGACAACAAGAGGCTGCTCGTGGAGGACGAGAAGCGTGCCGAGGACTACAA
GGGCGTGAAGAAGCTGCTCGACAGGTACTACCTGTCATTCATCAACGACGTCCTGCACTCAATCAAG
CTGAAGAACCTGAACAACTACATCTCACTGTTCCGTAAGAAGACCAGGACCGAGAAGGAGAACAAG
GAGCTGGAGAACCTGGAGATCAACCTGCGTAAGGAGATCGCCAAGGCTTTCAAGGGCAACGAGGG
CTACAAGTCACTGTTCAAGAAGGACATCATCGAGACAATCCTGCCAGAGTTCCTCGACGACAAGGAC
GAGATCGCCCTGGTCAACTCCTTCAACGGCTTCACCACCGCCTTCACTGGCTTCTTCAGGAACAGA
GAGAACATGTTCTCTGAGGAGGCCAAGTCCACCTCTATCGCCTTCAGGTGCATCAACGAGAACCTCA
CCCGTTACATCTCCAACATGGACATCTTCGAGAAGGTGGACGCCATCTTCGACAAGCACGAGGTGC
AGGAGATCAAGGAGAAGATCCTCAACTCCGACTACGACGTGGAGGACTTCTTCGAGGGTGAGTTCT
TCAACTTCGTGCTCACTCAGGAGGGTATCAGAGTGTACAACGCTATCATCGGTGGCTTCGTGACTGA
GTCCGGTGAGAAGATCAAGGGCCTGAACGAGTACATCAACCTGTACAACCAGAAGACCAAGCAGAA
GCTGCCAAAGTTCAAGCCTCTGTACAAGCAGGTCCTGTCAGACAGAGAGTCACTGTCATTCTACGG
CAGAGGCTACACTTCCGACGAGGAGGTCCTGGAGGTGTTCAGGAACACCCTCAACAAGAACTCCG
AGATCTTCTCTAGCATCAAGAAGCTCGAGAAGCTGTTCAAGAACTTCGACGAGTACTCTAGCGCTGG
CATCTTCGTGAAGAACGGACCTGCCATCTCCACCATCTCCAAGCGTATCTTCGGCGAGTGGAACGTG
//TACCACACTGAGCTACGACGTGTACAAGGACAAGAGGTTCTCCGAGGACCAGTACGAG//

Codons in red indicate the D156R, D235R, E292R, and D350R mutations. “//” means the sequence from ttLbCas12a Ultra V2 is omitted for brevity.

### Sequence of hyperCas12a Ultra

bpNLS$^{SV40}$-hyperCas12a Ultra-linker- bpNLS$^{SV40}$-linker- NLS$^{c-Myc}$

ATGAAGCGTACCGCTGATGGCAGCGAGTTCGAGAGCCCTAAGAAGAAGCGTAAGGTGTCAAAGCTC
GAGAAGTTCACCAACTGCTACTCCCTGAGCAAGACCCTGAGGTTCAAGGCCATCCCAGTCGGCAAG
ACCCAGGAGAACATCGACAACAAGAGGCTGCTCGTGGAGGACGAGAAGCGTGCCGAGGACTACAA
GGGCGTGAAGAAGCTGCTCGACAGGTACTACCTGTCATTCATCAACGACGTCCTGCACTCAATCAAG
CTGAAGAACCTGAACAACTACATCTCACTGTTCCGTAAGAAGACCAGGACCGAGAAGGAGAACAAG
GAGCTGGAGAACCTGGAGATCAACCTGCGTAAGGAGATCGCCAAGGCTTTCAAGGGCAACGAGGG
CTACAAGTCACTGTTCAAGAAGGACATCATCGAGACAATCCTGCCAGAGTTCCTCGACGACAAGGAC
GAGATCGCCCTGGTCAACTCCTTCAACGGCTTCACCACCGCCTTCACTGGCTTCTTCAGGAACAGA
GAGAACATGTTCTCTGAGGAGGCCAAGTCCACCTCTATCGCCTTCAGGTGCATCAACGAGAACCTCA
CCCGTTACATCTCCAACATGGACATCTTCGAGAAGGTGGACGCCATCTTCGACAAGCACGAGGTGC
AGGAGATCAAGGAGAAGATCCTCAACTCCGACTACGACGTGGAGGACTTCTTCGAGGGTGAGTTCT
TCAACTTCGTGCTCACTCAGGAGGGTATCAGAGTGTACAACGCTATCATCGGTGGCTTCGTGACTGA
GTCCGGTGAGAAGATCAAGGGCCTGAACGAGTACATCAACCTGTACAACCAGAAGACCAAGCAGAA
GCTGCCAAAGTTCAAGCCTCTGTACAAGCAGGTCCTGTCAGACAGAGAGTCACTGTCATTCTACGG
CAGAGGCTACACTTCCGACGAGGAGGTCCTGGAGGTGTTCAGGAACACCCTCAACAAGAACTCCG
AGATCTTCTCTAGCATCAAGAAGCTCGAGAAGCTGTTCAAGAACTTCGACGAGTACTCTAGCGCTGG
CATCTTCGTGAAGAACGGACCTGCCATCTCCACCATCTCCAAGCGTATCTTCGGCGAGTGGAACGTG
//TACCACACTGAGCTACGACGTGTACAAGGACAAGAGGTTCTCCGAGGACCAGTACCTG//

Codons in red indicate the D156R, D235R, E292R, D350R, and E795L mutations. “//” means the sequence from ttLbCas12a Ultra V2 is omitted for brevity.

### Sequence of Cas12i3V1

bpNLS$^{SV40}$-Cas12i3V1-linker- bpNLS$^{SV40}$-linker- NLS$^{c-Myc}$

ATGAAGCGCACCGCCGACGGCAGCGAGTTCGAGTCGCCCAAGAAGAAGCGCAAGGTGAAGAAGGT
CGAGGTGCGCAGGCCATACCAGAGCCTGCTCCTGCCAAACCATCGGAAGTTCAAGTACCTCGATGA
GACTTGGAATGCTTACAAGTCCGTCAAGAGCCTGCTCCATCGCTTCCTGGTGTGCGCTTACGGCGC
TGTGCCCTTCAACAAGTTCGTGGAGGTCGTCGAGAAGGTCGACAACGATCAGCTCGTGCTGGCGTT
CGCTGTGCGCCTGTTCCGCCTGGTGCCCGTGGAGAGCACCTCGTTCGCTAAGGTCGACAAGGCCA
ATCTGGCTAAGTCCCTGGCCAATCATCTGCCTGTGGGCACAGCCATTCCTGCCAATGTGCAGTCCTA
CTTCGATTCAAACTTCGATCCCAAGAAGTACATGTGGATCGACTGCGCGTGGGAGGCTGATCGCCTG
GCTCGGGAGATGGGCCTGAGCGCGAGCCAGTTCTCTGAGTACGCGACTACAATGCTCTGGGAGGA
CTGGCTGCCGCTCAACAAGGATGATGTGAACGGCTGGGGCTCCGTGTCCGGGCTCTTCGGCGAGG
GCAAGAAGGAGGACCGGCAGCAGAAGGTGAAGATGCTGAACAACCTGCTGAATGGCATCAAGAAG
AATCCACCCAAGGACTACACCCAGTACCTGAAGATCCTCCTGAACGCGTTCGACGCGAAGTCGCAC
AAGGAGGCTGTCAAGAACTACAAGGGCAGGTCCACTGGGCGCACCGCGTCGTACCTGTCCGAGAA
GTCTGGCGAGATCACCGAGCTGATGCTCGAGCAGCTGATGTCCAACATCCAGAGGGACATTGGCCG
CAAGCAGAAGGAGATCTCCCTGCCCAAGAAGGACGTGGTCAAGAAGTACCTCGAGTCAGAGTCTG
GCGTCCCGTACGACCAGAACCTGTGGTCCCAGGCGTACCGCAACGCTGCCAGCTCGATCAAGAAG
ACCGACACGCGGAACTTCAACTCCACGCTCGAGAAGTTCAAGAATGAGGTGGAGCTGCGCGGCCT
GCTGAGCGAGGGCGACGACGTCGAGATCCTGAGGTCCAAGTTCTTCAGCAGCGAGTTCCACAAGA
CGCCTGACAAGTTCGTCATCAAGCCAGAGCACATCGGGTTCAACCGCAAGTACAATGTCGTCGCCG
AGCTGTACAAGCTCAAGGCCGAGGCGACCGACTTCGAGAGCGCGTTCGCCACCGTCAAGGACGAG
TTCGAGGAGAAGGGCATCAAGCATCCAATCAAGAACATCCTCGAGTACATCTGGAACAACGAGGTGC
CTGTCGAGAAGTGGGGCAGGGTCGCTCGCTTCAATCAGCGCGAGGAGAAGCTCCTCCGGATCAAG
GCCAATCCTACGGTGGAGTGCAATCAGGGCATGACCTTCGGCAACAGCGCTATGGTCGGCGAGGTC
CTCAGGTCCAACTACGTCTCTAAGAAGGGCGCTCTGGTGTCCGGCGAGCATGGCGGCAGGCTGAT
CGGCCAGAACAACATGATCTGGCTGGAGATGCGGCTGCTCAACAAGGGCAAGTGGAGACGCACC
ACGTGCCAACCCACAACATGAAGTTCTTCGAGGAGGTGCACGCCTACAATCCGTCGCTGGCGGACT
CCGTCAACGTGCGGAATCGGCTGTACCGCTCCGAGGACTACACCCAGCTGCCTAGCAGCATCACCG
ACGGGCTGAAGGGCAATCCGAAGGCGAAGCTCCTGAAGCGCCAGCACTGCGCTCTGAACAACATG
ACAGCCAATGTGCTCAATCCCAAGCTGAGCTTCACGATCAACAAGAAGAACGACGACTACACGGTCA
TCATTGTCCACAGCGTCGAGGTGTCGAAGCCCAGGCGCGAGGTGCTCGTCGGCGACTACCTCGTG
GGCATGGACCAGAATCAGACTGCGTCTAACACATACGCCGTCATGCAGGTCGTCAAGCCTAAGTCTA
CCGATGCGATCCCGTTCCGCAACATGTGGGTGCGGTTCGTGGAGTCAGGGTCTATCGAGTCCCGGA
CACTCAACTCACGCGGCGAGTATGTCGATCAGCTGAATCATGATGGCGTGGACCTCTTCGAGATCG
GCGATACTGAGTGGGTGGACTCCGCTCGCAAGTTCTTCAACAAGCTCGGCGTCAAGCACAAGGATG
GCACACTGGTCGATCTGTCTACTGCGCCACGCAAGGCGTACGCTTTCAACAACTTCTACTTCAAGAC
CATGCTGAATCATCTCCGGAGCAATGAGGTCGACCTGACGCTCCTGCGCAATGAGATCCTCCGGGT
CGCCAATGGGCGGTTCTCACCGATGCGCCTCGGCTCACTCTCCTGGACTACTCTCAAGGCCCTGGG
CTCGTTCAAGTCCCTGGTGCTGTCGTACTTCGACCGGCTGGGCGCCAAGGAGATGGTCGACAAGG
AGGCTAAGGACAAGTCACTGTTCGACCTCCTCGTGGCTATCAACAACAAGCGCTCTAACAAGCGCG
AGGAGCGGACTTCCCGGATTGCCTCCAGCCTCATGACTGTGGCGCAGAAGTACAAGGTCGACAAC
GCTGTGGTCCATGTGGTCGTCGAGGGCAATCTCTCCAGCACGGACAGGAGCGCGTCAAAGGCCCA
CAATCGGAACACTATGGATTGGTGCTCTAGGGCCGTGGTGAAGAAGCTGGAGGACATGTGCAATCT
CTACGGCTTCAACATCAAGGGCGTCCCAGCCTTCTACACATCCCACCAGGACCCGCTCGTCCACCG
CGCTGACTACGATGATCCTAAGCCTGCGCTCAGGTGCCGCTACTCATCGTACTCAAGGGCTGACTTC
AGCAAGTGGGGCCAGAATGCTCTCGCTGCTGTGGTGCGCTGGGCGTCTAACAAGAAGTCCAACAC

ATGCTACAAGGTCGGCGCTGTGGAGTTCCTCAAGCAGCATGGCCTCTTCGCTGACAAGAAGCTGAC
AGTCGAGCAGTTCCTGTCTAAGGTGAAGGATGAGGAGATCCTCATTCCCAGGCGCGGCGGCAGGG
TGTTCCTCACAACTCATCGGCTCCTGGCTGAGTCCACGTTCGTGTACCTGAACGGCGTCAAGTACCA
TTCGTGCAACGCCGACGAGGTGGCGGCTGTCAACATCTGCCTGAACGACTGGGTCATCCCGTGCA
AGAAGAAGATGAAGGAGGAGTCCAGCGCGTCTGGGTCTGGCGGCTCTAAGCGCACTGCTGATGGC
TCTGAGTTCGAGTCACCTAAGAAGAAGCGCAAGGTCGGCTCTGGCCCTGCTGCTAAGCGCGTCAAG
CTCGACTGA

Codons in red indicate the S7R, D233R, D267R, N369R, and S433R mutations.

## Sequence of Cas12i3V2

bpNLS$^{SV40}$-Cas12i3V2-linker- bpNLS$^{SV40}$-linker- NLS$^{c-Myc}$

ATGAAGCGTACCGCTGACGGCAGCGAGTTCGAGAGCCCTAAGAAGAAGCGTAAGGTCAAGAAGGT
CGAGGTGCGTAGGCCATACCAGTCACTGCTCCTGCCTAACCACAGGAAGTTCAAGTACCTCGACGA
GACTTGGAACGCTTACAAGTCTGTCAAGAGCCTGCTCCACAGATTCCTGGTGTGCGCTTACGGTGC
TGTGCCATTCAACAAGTTCGTGGAGGTCGTCGAGAAGGTCGACAACGACCAGCTCGTGCTGGCCTT
CGCTGTGAGACTGTTCAGACTGGTGCCCGTCGAGTCCACATCATTCGCTAAGGTCGACAAGGCCAA
CCTGGCTAAGTCCCTGGCCAACCACCTCCCTGTGGGCACAGCCATCCCTGCCAACGTGCAGTCCTA
CTTCGACAGCAACTTCGACCCTAAGAAGTACATGTGGATCGACTGCGCTTGGGAGGCTGACAGACT
GGCTCGTGAGATGGGCCTGAGCGCTAGCCAGTTCAGCGAGTACGCTACTACAATGCTCTGGGAGGA
CTGGCTGCCACTCAACAAGGACGACGTGAACGGCTGGGGCTCCGTGTCTGGCCTGTTCGGCGAGG
GCAAGAAGGAGGACAGACAGCAGAAGGTGAAGATGCTGAACAACCTGCTGAACGGCATCAAGAAG
AACCCACCCAAGGACTACACCCAGTACCTGAAGATCCTGCTGAACGCCTTCGACGCTAAGTCCCAC
AAGGAGGCTGTCAAGAACTACAAGGGCAGGTCCACTGGCAGAACCGCTAGCTACCTGTCCGAGAA
GTCTGGCGAGATCACCGAGCTGATGCTCGAGCAGCTGATGTCCAACATCCAGCGTGACATCGGCCG
TAAGCAGAAGGAGATCTCACTGCCCAAGAAGGACGTGGTCAAGAAGTACCTCGAGTCAGAGTCTGG
CGTCCCATACGACCAGAACCTGTGGTCCCAGGCTTACCGTAACGCTGCCAGCTCCATCAAGAAGAC
CGACACCCGTAACTTCAACTCCACCCTCGAGAAGTTCAAGAACGAGGTGGAGCTGCGTGGTCTGCT
GAGCGAGGGCGACGACGTCGAGATCCTGAGGTCCAAGTTCTTCAGCAGCGAGTTCCACAAGACAC
CTGACAAGTTCGTCATCAAGCCAGAGCACATCGGCTTCAACAGGAAGTACAACGTCGTCGCCGAGC
TGTACAAGCTCAAGGCCGAGGCTACCGACTTCGAGTCAGCCTTCGCCACCGTCAAGGACGAGTTCG
AGGAGAAGGGCATCAAGCACCCAATCAAGAACATCCTCGAGTACATCTGGAACAACGAGGTGCCTG
TCGAGAAGTGGGGCAGGGTCGCTAGATTCAACCAGCGTGAGGAGAAGCTGCTCAGAATCAAGGCC
AACCCTACCGTGGAGTGCAACCAGGGCATGACCTTCGGCAACAGCGCTATGGTCGGCGAGGTCCT
CAGGTCCAACTACGTCAGCAAGAAGGGTGCTCTGGTGTCTGGCGAGCACGGTGGCAGGCTGATCG
GCCAGAACAACATGATCTGGCTGGAGATGAGACTGCTCAACAAGGGCAAGTGGGAGACACACCAC
GTGCCAACCCACAACATGAAGTTCTTCGAGGAGGTGCACGCCTACAACCCATCCCTGGCTGACTCC
GTCAACGTGCGTAACAGACTGTACAGATCCGAGGACTACACCCAGCTGCCTAGCAGCATCACCGAC
GGCCTCAAGGGCAACCCTAAGGCTAAGCTCCTGAAGCGTCAGCACTGCGCTCTGAACAACATGACA
GCCAACGTGCTCAACCCTAAGCTGAGCTTCACCATCAACAAGAAGAACGACGACTACACCGTCATCA
TCGTCCACAGCGTCGAGGTGTCCAAGCCCAGGCGTGAGGTGCTCGTCGGCGACTACCTCGTGGGC
ATGGACCAGAACCAGACTGCTTCTAACACATACGCCGTCATGCAGGTCGTCAAGCCTAAGTCTACCG
ACGCTATCCCATTCCGTAACATGTGGGTGAGATTCGTGGAGTCAGGCTCTATCGAGTCCAGAACACT
CAACTCACGTGGCGAGTATGTCGACCAGCTGAACCACGACGGCGTGGACCTGTTCGAGATCGGCG
ACACTGAGTGGGTGGACTCCGCTAGGAAGTTCTTCAACAAGCTCGGCGTCAAGCACAAGGACGGC

ACACTGGTCGACCTGTCTACTGCTCCACGTAAGGCTTACGCCTTCAACAACTTCTACTTCAAGACCAT
GCTGAACCACCTCAGAAGCAACGAGGTCGACCTGACCCTGCTGCGTAACGAGATCCTCAGAGTCG
CCAACGGCAGATTCTCACCAATGAGACTCGGCTCACTGTCCTGGACTACTCTCAAGGCCCTGGGCA
GCTTCAAGTCCCTGGTGCTGTCCTACTTCGACAGACTCGGTGCTAAGGAGATGGTCGACAAGGAGG
CTAAGGACAAGTCACTGTTCGACCTGCTCGTGGCTATCAACAACAAGCGTTCTAACAAGCGTGAGGA
GCGTACTTCCAGAATCGCCTCCAGCCTCATGACTGTGGCTCAGAAGTACAAGGTCGACAACGCTGT
GGTCCACGTGGTCGTCGAGGGCAACCTGTCCAGCACCGACAGGAGCGCTAGCAAGGCCCACAACC
GTAACACTATGGACTGGTGCTCTAGGGCTGTCGTGAAGAAGCTCGAGGACATGTGCAACCTCTACG
GCTTCAACATCAAGGGCGTCCCAGCCTTCTACACATCCCACCAGGACCCACTCGTCCACAGAGCTG
ACTACGACGACCCTAAGCCTGCTCTCAGGTGCAGATACTCATCCTACTCACGTGCTGACTTCAGCAA
GTGGGGTCAGAACGCTCTCGCTGCTGTGGTGAGATGGGCTTCTAACAAGAAGTCCAACACATGCTA
CAAGGTCGGTGCTGTGGAGTTCCTCAAGCAGCACGGCCTGTTCGCTGACAAGAAGCTGACAGTCG
AGCAGTTCCTGTCTAAGGTCAAGGACGAGGAGATCCTCATCCCACGTAGAGGTGGCAGGGTGTTCC
TGACAACTCACAGACTCCTGGCTGAGTCCACCTTCGTGTACCTGAACGGCGTCAAGTACCACTCCT
GCAACGCCGACGAGGTGGCTGCTGTCAACATCTGCCTGAACGACTGGGTCATCCCATGCAAGAAGA
AGATGAAGGAGGAGTCCAGCGCTTCTGGCTCTGGTGGATCTAAGAGGACTGCCGACGGATCTGAGT
TCGAGTCACCCAAGAAGAAGCGTAAGGTCGGATCTGGACCTGCTGCTAAGAGAGTCAAGCTCGACT
GA

Codons in red indicate the S7R, D233R, D267R, N369R, and S433R mutations.

**Sequence of AsCas12f-YHAM**

bpNLS^SV40-Cas12f-YHAM-linker- bpNLS^SV40-linker- NLS^c-Myc

ATGAAGCGTACCGCTGACGGCAGCGAGTTCGAGAGCCCTAAGAAGAAGCGTAAGGTCATCAAGGTG
TACAGATACGAGATCGTCAAGCCTCTGGACCTCGACTGGAAGGAGTTCGGCACCATCCTGAGACAG
CTGCAGCAGGAGACTAGATTCGCTCTCAACAAGGCCACACAGCTCGCCTGGGAGTGGATGGGCTAC
AGCAGCGACTACAAGGACAACCACGGCGAGTACCCTAAGAGCAAGGACATCCTCGGCTACACCAAC
GTGCACGGCTACGCCTACCACACCATCAAGACTAAGGCTTACAGACTCAACTCTGGCAACCTGTCAC
AGACCATCAAGCGTGCCACAGACAGGTTCAAGGCTTACCAGAAGGAGATCCTGCGTGGCGACATGT
CTATCCCTAGCTACAAGCGTGACATCCCACTCGACCTGATCAAGGAGAACATCTCCGTGAACAGGAT
GAACCACGGCGACTACATCGCTAGCCTGTCACTGCTCAGCAACCCTGCCAAGCAGGAGATGAACGT
CAAGCGTAAGATCTCCGTGATCATCATCGTGCGTGGCGCTGGCAAGACCATCATGGACAGAATCCTG
TCTGGCGAGTACCAGGTGCATGCCAGCCAGATCATCCACGACGACCGTAAGAACAAGTGGTACCTG
AACATCAGCTACGACTTCGAGCCACAGACCAGAGTGCTCGACCTGAACAAGATCATGGGCATCGAC
CTCGGCGTGGCTGTGGCTGCTTACATGGCCTTCCAGCACACACCCGCTAGGTACAAGCTGGAGGGT
GGCGAGATCGAGAACTTCAGGAGGCAGGTCGAGAGCAGACGTATCTCCATGCTCAGACAGGGCAA
GTACGCTGGTGGTGCTAGAGGTGGTCACGGCAGAGACAAGCGTATCAAGCCCATCGAGCAGCTGC
GTGACAAGATCGCCAACTTCAGAGACACCACCAACCACAGATACAGCAGATACATCGTGGACATGGC
CATCAAGATGGGCTGCGGCACAATCCAGATGGAGGATCTGACTAACATCAGAGACATCGGCAGCAG
ATTCCTGCAGAACTGGACCTACTACGACCTGCAGCAGAAGATCATCTACAAGGCTGAGGAGGCTGG
CATCAAGGTCATCAAGATCGACCCACAGTACACCAGCCAGAGATGCTCCGAGTGCGGCAACATCGA
CAGCGGCAACAGAATCGGCCAGGCCATCTTCAAGTGCAGAGCCTGCGGCTACGAGGCCAACGCTG
ACTACAACGCTGCCAGGAACATCGCCATCCCTAACATCGACAAGATCATCGCTGAGAGCATCAAGTC
TGGTGGATCTAAGAGGACTGCCGACGGATCTGAGTTCGAGTCACCCAAGAAGAAGCGTAAGGTCGG
ATCTGGACCTGCTGCTAAGAGAGTCAAGCTCGACTGA

Codons in red indicate the F48Y, S188H, V232A, and E316M mutations.

### Sequence of AsCas12f-HKRA

bpNLS$^{SV40}$-AsCas12f-HKRA-linker- bpNLS$^{SV40}$-linker- NLS$^{c-Myc}$

ATGAAGCGTACCGCTGACGGCAGCGAGTTCGAGAGCCCTAAGAAGAAGCGTAAGGTCATCAAGGTG
TACAGATACGAGATCGTCAAGCCTCTGGACCTCGACTGGAAGGAGTTCGGCACCATCCTGAGACAG
CTGCAGCAGGAGACTAGATTCGCTCTCAACAAGGCCACACAGCTCGCCTGGGAGTGGATGGGCTTC
AGCAGCGACTACAAGGACAACCACGGCGAGTACCCTAAGAGCAAGGACATCCTCGGCTACACCAAC
GTGCACGGCTACGCCTACCACACCATCAAGACTAAGGCTTACAGACTCAACTCTGGCAACCTGTCAC
AGACCATCAAGCGTGCCACAGACAGGTTCAAGGCTTACCAGAAGGAGATCCTGCGTGGCGACATGT
CTATCCCTAGCTACAAGCGTGACCATCCACTCGACCTGATCAAGGAGAACATCTCCGTGAACAGGAT
GAACCACGGCGACTACATCGCTAGCCTGTCACTGCTCAGCAACCCTGCCAAGCAGGAGATGAACGT
CAAGCGTAAGATCTCCGTGATCATCATCGTGCGTGGCGCTGGCAAGACCATCATGGACAGAATCCTG
TCTGGCGAGTACCAGGTGTCCGCCAGCCAGATCATCCACAAGGACCGTAAGAACAAGTGGTACCTG
AACATCAGCTACAGGTTCGAGCCACAGACCAGAGTGCTCGACCTGAACAAGATCATGGGCATCGAC
CTCGGCGTGGCTGTGGCTGCTTACATGGCCTTCCAGCACACACCCGCTAGGTACAAGCTGGAGGGT
GGCGAGATCGAGAACTTCAGGAGGCAGGTCGAGAGCAGACGTATCTCCATGCTCAGACAGGGCAA
GTACGCTGGTGGTGCTAGAGGTGGTCACGGCAGAGACAAGCGTATCAAGCCCATCGAGCAGCTGC
GTGACAAGATCGCCAACTTCAGAGACACCACCAACCACAGATACAGCAGATACATCGTGGACATGGC
CATCAAGGAGGGCTGCGGCACAATCCAGATGGAGGATCTGACTAACATCAGAGACATCGGCAGCAG
ATTCCTGCAGAACTGGACCTACTACGACCTGCAGCAGAAGATCATCTACAAGGCTGAGGAGGCTGG
CATCAAGGTCATCAAGATCGACCCACAGTACACCAGCCAGAGATGCTCCGAGTGCGGCAACATCGA
CAGCGGCAACAGAATCGGCCAGGCCATCTTCAAGTGCAGAGCCTGCGGCTACGAGGCCAACGCTG
ACTACAACGCTGCCAGGAACATCGCCATCCCTAACATCGACAAGATCATCGCTGAGAGCATCAAGTC
TGGTGGATCTAAGAGGACTGCCGACGGATCTGAGTTCGAGTCACCCAAGAAGAAGCGTAAGGTCGG
ATCTGGACCTGCTGCTAAGAGAGTCAAGCTCGACTGA

Codons in red indicate the I123H, D195K, D208R, and V232A mutations.

## Cloning cassette for assembly of LbCas12a crRNA

U6-26p-DR-BsaI-BsaI-HDVd1-U6-26t

CGACTTGCCTTCCGCACAATACATCATTTCTTCTTAGCTTTTTTTCTTCTTCTTCGTTCATACAGTTTTT
TTTTGTTTATCAGCTTACATTTTCTTGAACCGTAGCTTTCGTTTTCTTCTTTTTAACTTTCCATTCGGAG
TTTTTGTATCTTGTTTCATAGTTTGTCCCAGGATTAGAATGATTAGGCATCGAACCTTCAAGAATTTGAT
TGAATAAAACATCTTCATTCTTAAGATATGAAGATAATCTTCAAAAGGCCCCTGGGAATCTGAAAGAAG
AGAAGCAGGCCCATTTATATGGGAAAGAACAATAGTATTTCTTATATAGGCCCATTTAAGTTGAAAACA
ATCTTCAAAAGTCCCACATCGCTTAGATAAGAAAACGAAGCTGAGTTTATATACAGCTAGAGTCGAAG
TAGTGATTGTAATTTCTACTAAGTGTAGATAGAGACCNNNNNNNNNNNNNNNNNNNNNNNNGGTCTCA
GCCGGCATGGTCCCAGCCTCCTCGCTGGCGCCGGCTGGGCAACATGCTTCGGCATGGCGAATGGG
ACTTTTTTTTGCAAAATTTTCCAGATCGATTTCTTCTTCCTCTGTTCTTCGGCGTTCAATTTCTGGGGT
TTTCTCTTCGTTTTCTGTAACTGAAACCTAAAATTTGACCTAAAAAAAATCTCAAATAATATGATTCAGT
GGTTTTGTACTTTTCAGTTAGTTGAGTTTTGCAGTTCCGATGAGATAAACCAATA

## Final cassette for expression of 1×crRNA for LbCas12a

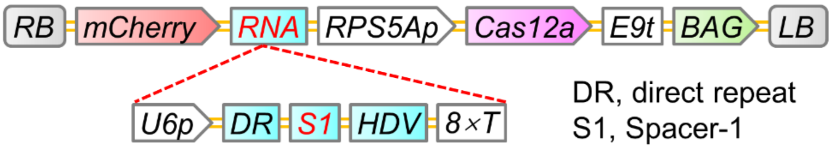


DR, direct repeat
S1, Spacer-1

U6-26p-DR-S1-HDV-U6-26t

CGACTTGCCTTCCGCACAATACATCATTTCTTCTTAGCTTTTTTTCTTCTTCTTCGTTCATACAGTTTTT
TTTTGTTTATCAGCTTACATTTTCTTGAACCGTAGCTTTCGTTTTCTTCTTTTTAACTTTCCATTCGGAG
TTTTTGTATCTTGTTTCATAGTTTGTCCCAGGATTAGAATGATTAGGCATCGAACCTTCAAGAATTTGAT
TGAATAAAACATCTTCATTCTTAAGATATGAAGATAATCTTCAAAAGGCCCCTGGGAATCTGAAAGAAG
AGAAGCAGGCCCATTTATATGGGAAAGAACAATAGTATTTCTTATATAGGCCCATTTAAGTTGAAAACA
ATCTTCAAAAGTCCCACATCGCTTAGATAAGAAAACGAAGCTGAGTTTATATACAGCTAGAGTCGAAG
TAGTGATTGTAATTTCTACTAAGTGTAGATNNNNNNNNNNNNNNNNNNNNNNNNGGCCGGCATGGTC
CCAGCCTCCTCGCTGGCGCCGGCTGGGCAACATGCTTCGGCATGGCGAATGGGACTTTTTTTTGCA
AAATTTTCCAGATCGATTTCTTCTTCCTCTGTTCTTCGGCGTTCAATTTCTGGGGTTTTCTCTTCGTTT
TCTGTAACTGAAACCTAAAATTTGACCTAAAAAAAATCTCAAATAATATGATTCAGTGGTTTTGTACTTT
TCAGTTAGTTGAGTTTTGCAGTTCCGATGAGATAAACCAATA

**Primers**

| | |
|---|---|
| oS1-F | **AGAT**NNNNNNNNNNNNNNNNNNNNNNNNG |
| oS1-R | **CGGC**CNNNNNNNNNNNNNNNNNNNNNNNN |

**Generation of short inserts by annealing two 5'-phosphorylated oligos (primers)**

oS1-F + R = Ins1-S1

**BsaI-based Golden Gate Cloning**

pBG-ttLbUV2 + Ins1-S1= **pBG-S1**

**Colony PCR and sequencing primers**

| | |
|---|---|
| U6-26p-F | TGTCCCAGGATTAGAATGATTAGGC |
| U6-26t-R | CCCCAGAAATTGAACGCCGAAGAAC |
| U6-26p-F + U6-26t-R = 440-bp | |

## Final cassette for expression of 2×crRNAs for LbCas12a

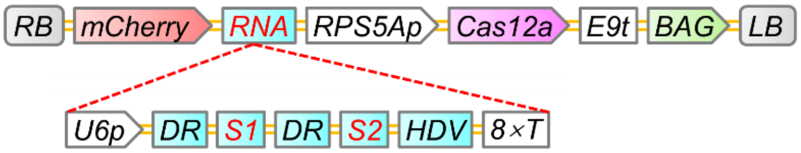


U6-26p-DR-S1-DR-S2-HDV-U6-26t

CGACTTGCCTTCCGCACAATACATCATTTCTTCTTAGCTTTTTTTCTTCTTCTTCGTTCATACAGTTTTT
TTTTGTTTATCAGCTTACATTTTCTTGAACCGTAGCTTTCGTTTTCTTCTTTTTAACTTTCCATTCGGAG
TTTTTGTATCTTGTTTCATAGTTTGTCCCAGGATTAGAATGATTAGGCATCGAACCTTCAAGAATTTGAT
TGAATAAAACATCTTCATTCTTAAGATATGAAGATAATCTTCAAAAGGCCCCTGGGAATCTGAAAGAAG
AGAAGCAGGCCCATTTATATGGGAAAGAACAATAGTATTTCTTATATAGGCCCATTTAAGTTGAAAACA
ATCTTCAAAAGTCCCACATCGCTTAGATAAGAAAACGAAGCTGAGTTTATATACAGCTAGAGTCGAAG
TAGTGATTGTAATTTCTACTAAGTGTAGATNNNNNNNNNNNNNNNNNNNNNNNNTAATTTCTACTAAGT
GTAGATNNNNNNNNNNNNNNNNNNNNNNNNGGCCGGCATGGTCCCAGCCTCCTCGCTGGCGCCGG
CTGGGCAACATGCTTCGGCATGGCGAATGGGACTTTTTTTTGCAAAATTTTCCAGATCGATTTCTTCT
TCCTCTGTTCTTCGGCGTTCAATTTCTGGGGTTTTCTCTTCGTTTTCTGTAACTGAAACCTAAAATTTG
ACCTAAAAAAAATCTCAAATAATATGATTCAGTGGTTTTGTACTTTTCAGTTAGTTGAGTTTTGCAGTTC
CGATGAGATAAACCAATA

**Primers**

| | | |
|---|---|---|
| oS1-F | **AGAT**NNNNNNNNNNNNNNNNNNNNNNNNTAATTT | 5'-Phosphorylation |
| oS1-R | **GTAG**AAATTANNNNNNNNNNNNNNNNNNNNNNNN | 5'-Phosphorylation |
| oS2-F | **CTAC**TAAGTGTAGATNNNNNNNNNNNNNNNNNNNNNNNNG | 5'-Phosphorylation |
| oS2-R | **CGGC**CNNNNNNNNNNNNNNNNNNNNNNNNATCTACACTTA | 5'-Phosphorylation |

**Generation of short inserts by annealing two 5'-phosphorylated oligos (primers)**

oS1-F + R = Ins-S1

oS2-F + R = Ins-S2

**BsaI-based Golden Gate Cloning**

pBG-ttLbUV2 + Ins-S1+ Ins-S2 = **pBG-S1&S2**

**Colony PCR and sequencing primers**

| | |
|---|---|
| U6-26p-F | TGTCCCAGGATTAGAATGATTAGGC |
| U6-26t-R | CCCCAGAAATTGAACGCCGAAGAAC |
| U6-26p-F + U6-26t-R = 484-bp | |

## Final cassette for expression of 3×crRNAs for LbCas12a

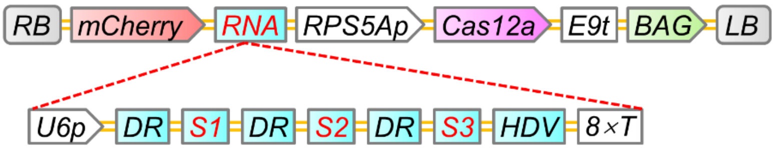


U6-26p-DR-S1-DR-S2-DR-S3-HDV-U6-26t

CGACTTGCCTTCCGCACAATACATCATTTCTTCTTAGCTTTTTTTCTTCTTCTTCGTTCATACAGTTTTT
TTTTGTTTATCAGCTTACATTTTCTTGAACCGTAGCTTTCGTTTTCTTCTTTTTAACTTTCCATTCGGAG
TTTTTGTATCTTGTTTCATAGTTTGTCCCAGGATTAGAATGATTAGGCATCGAACCTTCAAGAATTTGAT
TGAATAAAACATCTTCATTCTTAAGATATGAAGATAATCTTCAAAAGGCCCCTGGGAATCTGAAAGAAG
AGAAGCAGGCCCATTTATATGGGAAAGAACAATAGTATTTCTTATATAGGCCCATTTAAGTTGAAAACA
ATCTTCAAAAGTCCCACATCGCTTAGATAAGAAAACGAAGCTGAGTTTATATACAGCTAGAGTCGAAG
TAGTGATTGTAATTTCTACTAAGTGTAGATNNNNNNNNNNNNNNNNNNNNNNNTAATTTCTACTAAGT
GTAGATNNNNNNNNNNNNNNNNNNNNNNNTAATTTCTACTAAGTGTAGATNNNNNNNNNNNNNNNN
NNNNNNNGGCCGGCATGGTCCCAGCCTCCTCGCTGGCGCCGGCTGGGCAACATGCTTCGGCATG
GCGAATGGGACTTTTTTTTGCAAAATTTTCCAGATCGATTTCTTCTTCCTCTGTTCTTCGGCGTTCAAT
TTCTGGGGTTTTCTCTTCGTTTTCTGTAACTGAAACCTAAAATTTGACCTAAAAAAAATCTCAAATAATA
TGATTCAGTGGTTTTGTACTTTTCAGTTAGTTGAGTTTTGCAGTTCCGATGAGATAAACCAATA

**Primers**

| | | |
|---|---|---|
| oS1-F | **AGAT**NNNNNNNNNNNNNNNNNNNNNNNTAATTT | 5'-Phosphorylation |
| oS1-R | **GTAG**AAATTANNNNNNNNNNNNNNNNNNNNNNN | 5'-Phosphorylation |
| oS2-F | **CTAC**TAAGTGTAGATNNNNNNNNNNNNNNNNNNNNNNNTAATTTCTACT | 5'-Phosphorylation |
| oS2-R | **ACTT**AGTAGAAATTANNNNNNNNNNNNNNNNNNNNNNNATCTACACTTA | 5'-Phosphorylation |
| oS3-F | **AAGT**GTAGATNNNNNNNNNNNNNNNNNNNNNNNG | 5'-Phosphorylation |
| oS3-R | **CGGC**CNNNNNNNNNNNNNNNNNNNNNNNATCTAC | 5'-Phosphorylation |

**Generation of short inserts by annealing two 5'-phosphorylated oligos (primers)**

oS1-F + R = Ins-S1

oS2-F + R = Ins-S2

oS3-F + R = Ins-S3

**BsaI-based Golden Gate Cloning**

pBG-ttLbUV2 + Ins-S1 + Ins-S2 + Ins-S3 = **pBG-S1–S3**

**Colony PCR and sequencing primers**

| | |
|---|---|
| U6-26p-F | TGTCCCAGGATTAGAATGATTAGGC |
| U6-26t-R | CCCCAGAAATTGAACGCCGAAGAAC |
| U6-26p-F + U6-26t-R = 528-bp | |

## Cloning cassette in pAGC-Lb12-P1Bb

BsaI-BbsI-BbsI-HDV-U6-29t-U6-29p- DRd1-BbsI-BbsI-BsaI

TTCGCTACCTTAGGACCGTTATAGTTACGGTCTCAAGATATGTCTTCACAACCAATGAAGACAGTTGG
CCGGCATGGTCCCAGCCTCCTCGCTGGCGCCGGCTGGGCAACATGCTTCGGCATGGCGAATGGGA
CTTTTTTTTGGATAGAATTTCCCAGCTTTTTTGCGTGTTTCAGCTCTCATGATCCTTAATCCAAACTACT
GCAGCCTGACAGACAAATGAGGATGCAAACAATTTTAAAGTTTATCTAACGCTAGCTGTTTTGTTTCTT
CTCTCTGGTGCACCAACGACGGCGTTTTCTCAATCATAAAGAGGCTTGTTTTACTTAAGGCCAATAAT
GTTGATGGATCGAAAGAAGAGGGCTTTTAATAAACGAGCCCGTTTAAGCTGTAAACGATGTCAAAAAC
ATCCCACATCGTTCAGTTGAAAATAGAAGCTCTGTTTATATATTGGTAGAGTCGACTAAGAGATTGTAAT
TTCTACTAAGTGTAGAATGTCTTCATGGTGGTGTGAAGACATGCCGTGAGACCAATAGGGATAACAGG
GTAATGTATGGT

## Cloned sequence in pAGC-S1–S4

BsaI-S1-DR-S2-HDV-U6-29t-U6-29p-DR-S3-DR-S4-BsaI

TTCGCTACCTTAGGACCGTTATAGTTACGGTCTCAAGATNNNNNNNNNNNNNNNNNNNNNNNNTAAT
TTCTACTAAGTGTAGATNNNNNNNNNNNNNNNNNNNNNNNNTTGGCCGGCATGGTCCCAGCCTCCTC
GCTGGCGCCGGCTGGGCAACATGCTTCGGCATGGCGAATGGGACTTTTTTTTGGATAGAATTTCCCA
GCTTTTTTGCGTGTTTCAGCTCTCATGATCCTTAATCCAAACTACTGCAGCCTGACAGACAAATGAGG
ATGCAAACAATTTTAAAGTTTATCTAACGCTAGCTGTTTTGTTTCTTCTCTCTGGTGCACCAACGACGG
CGTTTTCTCAATCATAAAGAGGCTTGTTTTACTTAAGGCCAATAATGTTGATGGATCGAAAGAAGAGG
GCTTTTAATAAACGAGCCCGTTTAAGCTGTAAACGATGTCAAAAACATCCCACATCGTTCAGTTGAAA
ATAGAAGCTCTGTTTATATATTGGTAGAGTCGACTAAGAGATTGTAATTTCTACTAAGTGTAGATNNNN
NNNNNNNNNNNNNNNNNNNNTAATTTCTACTAAGTGTAGATNNNNNNNNNNNNNNNNNNNNNNNNGG
CCGTGAGACCAATAGGGATAACAGGGTAATGTATGGT

(Two extra T bases in S2 do not affect editing efficiency)

**Primers**

| | | |
|---|---|---|
| oS1-F | **AGAT**NNNNNNNNNNNNNNNNNNNNNNNNTAAT | 5'-Phosphorylation |
| oS1-R | **AGAA**ATTANNNNNNNNNNNNNNNNNNNNNNNN | 5'-Phosphorylation |
| oS2-F | **TTCT**ACTAAGTGTAGATNNNNNNNNNNNNNNNNNNNNNNNN | 5'-Phosphorylation |
| oS2-R | **CCAA**NNNNNNNNNNNNNNNNNNNNNNNNATCTACACTTAGT | 5'-Phosphorylation |
| oS3-F | **TAGA**TNNNNNNNNNNNNNNNNNNNNNNNNTAATTTCTACT | 5'-Phosphorylation |
| oS3-R | **ACTT**AGTAGAAATTANNNNNNNNNNNNNNNNNNNNNNNNA | 5'-Phosphorylation |
| oS4-F | **AAGT**GTAGATNNNNNNNNNNNNNNNNNNNNNNNNG | 5'-Phosphorylation |
| oS4-R | **CGGC**CNNNNNNNNNNNNNNNNNNNNNNNNATCTAC | 5'-Phosphorylation |

**Generation of short inserts by annealing two 5'-phosphorylated oligos (primers)**

oS1-F + R = Ins-S1

oS2-F + R = Ins-S2

oS3-F + R = Ins-S3

oS4-F + R = Ins-S4

**BbsI-based Golden Gate Cloning**

pAGC-Lb12-P1Bb + Ins-S1 + Ins-S2 + Ins-S3 + Ins-S4 = **pAGC-S1–S4**

**Colony PCR primers:** oS1-F + oS4-R = 574-bp

**Sequencing primers:** M13F and M13R

## Final cassette for expression of 4×crRNAs for LbCas12a

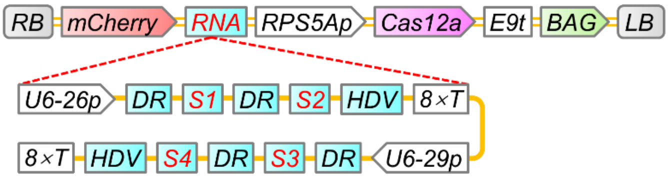


U6-26p-DR-S1-DR-S2-HDV-U6-29t-U6-29p-DR-S3-DR-S4-HDV-U6-26t

CGACTTGCCTTCCGCACAATACATCATTTCTTCTTAGCTTTTTTTCTTCTTCTTCGTTCATACAGTTTTT
TTTTGTTTATCAGCTTACATTTTCTTGAACCGTAGCTTTCGTTTTCTTCTTTTTAACTTTCCATTCGGAG
TTTTTGTATCTTGTTTCATAGTTTGTCCCAGGATTAGAATGATTAGGCATCGAACCTTCAAGAATTTGAT
TGAATAAAACATCTTCATTCTTAAGATATGAAGATAATCTTCAAAAGGCCCCTGGGAATCTGAAAGAAG
AGAAGCAGGCCCATTTATATGGGAAAGAACAATAGTATTTCTTATATAGGCCCATTTAAGTTGAAAACA
ATCTTCAAAAGTCCCACATCGCTTAGATAAGAAAACGAAGCTGAGTTTATATACAGCTAGAGTCGAAG
TAGTGATTGTAATTTCTACTAAGTGTAGATNNNNNNNNNNNNNNNNNNNNNNNTAATTTCTACTAAGTG
TAGATNNNNNNNNNNNNNNNNNNNNNNNTTGGCCGGCATGGTCCCAGCCTCCTCGCTGGCGCCGG
CTGGGCAACATGCTTCGGCATGGCGAATGGGACTTTTTTTTGGATAGAATTTCCCAGCTTTTTTGCGT
GTTTCAGCTCTCATGATCCTTAATCCAAACTACTGCAGCCTGACAGACAAATGAGGATGCAAACAATT
TTAAAGTTTATCTAACGCTAGCTGTTTTGTTTCTTCTCTCTGGTGCACCAACGACGGCGTTTTCTCAAT
CATAAAGAGGCTTGTTTTACTTAAGGCCAATAATGTTGATGGATCGAAAGAAGAGGGCTTTTAATAAAC
GAGCCCGTTTAAGCTGTAAACGATGTCAAAAACATCCCACATCGTTCAGTTGAAAATAGAAGCTCTGT
TTATATATTGGTAGAGTCGACTAAGAGATTGTAATTTCTACTAAGTGTAGATNNNNNNNNNNNNNNNNN
NNNNNNTAATTTCTACTAAGTGTAGATNNNNNNNNNNNNNNNNNNNNNNNGGCCGGCATGGTCCCA
GCCTCCTCGCTGGCGCCGGCTGGGCAACATGCTTCGGCATGGCGAATGGGACTTTTTTTTGCAAAA
TTTTCCAGATCGATTTCTTCTTCCTCTGTTCTTCGGCGTTCAATTTCTGGGGTTTTCTCTTCGTTTTCT
GTAACTGAAACCTAAAATTTGACCTAAAAAAAATCTCAAATAATATGATTCAGTGGTTTTGTACTTTTCA
GTTAGTTGAGTTTTGCAGTTCCGATGAGATAAACCAATA

(Two extra T bases in S2 do not affect editing efficiency)

**BsaI-based Golden Gate Cloning**

pBG-ttLbUV2 + pAGC-S1–S4 = **pBG-S1–S4**

**Colony PCR and sequencing primers**

| | |
|---|---|
| U6-26p-F | TGTCCCAGGATTAGAATGATTAGGC |
| U6-26t-R | CCCCAGAAATTGAACGCCGAAGAAC |
| U6-26p-F + U6-26t-R = 1.0-kb | |

## Cloned sequence in pAGC-S1–S6

BsaI-S1-DR-S2-DR-S3-HDV-U6-29t-U6-29p-DR-S4-DR-S5-DR-S6-BsaI

TTCGCTACCTTAGGACCGTTATAGTTACGGTCTCAAGATNNNNNNNNNNNNNNNNNNNNNNNTAAT
TTCTACTAAGTGTAGATNNNNNNNNNNNNNNNNNNNNNNNTAATTTCTACTAAGTGTAGATNNNNNN
NNNNNNNNNNNNNNNNNTTGGCCGGCATGGTCCCAGCCTCCTCGCTGGCGCCGGCTGGGCAACA
TGCTTCGGCATGGCGAATGGGACTTTTTTTTGGATAGAATTTCCCAGCTTTTTTGCGTGTTTCAGCTC
TCATGATCCTTAATCCAAACTACTGCAGCCTGACAGACAAATGAGGATGCAAACAATTTTAAAGTTTAT
CTAACGCTAGCTGTTTTGTTTCTTCTCTCTGGTGCACCAACGACGGCGTTTTCTCAATCATAAAGAGG
CTTGTTTTACTTAAGGCCAATAATGTTGATGGATCGAAAGAAGAGGGCTTTTAATAAACGAGCCCGTTT
AAGCTGTAAACGATGTCAAAAACATCCCACATCGTTCAGTTGAAAATAGAAGCTCTGTTTATATATTGG
TAGAGTCGACTAAGAGATTGTAATTTCTACTAAGTGTAGATNNNNNNNNNNNNNNNNNNNNNNNTAAT
TTCTACTAAGTGTAGATNNNNNNNNNNNNNNNNNNNNNNNTAATTTCTACTAAGTGTAGATNNNNNN
NNNNNNNNNNNNNNNNNGGCCGTGAGACCAATAGGGATAACAGGGTAATGTATGGT

(Two extra T bases in S3 do not affect editing efficiency)

**Primers**

| | | |
|---|---|---|
| oS1-F | **AGAT**NNNNNNNNNNNNNNNNNNNNNNNTAAT | 5'-Phosphorylation |
| oS1-R | **AGAA**ATTANNNNNNNNNNNNNNNNNNNNNNN | 5'-Phosphorylation |
| oS2-F | **TTCT**ACTAAGTGTAGATNNNNNNNNNNNNNNNNNNNNNNNTAATTT | 5'-Phosphorylation |
| oS2-R | **GTAG**AAATTANNNNNNNNNNNNNNNNNNNNNNNATCTACACTTAGT | 5'-Phosphorylation |
| oS3-F | **CTAC**TAAGTGTAGATNNNNNNNNNNNNNNNNNNNNNNN | 5'-Phosphorylation |
| oS3-R | **CCAA**NNNNNNNNNNNNNNNNNNNNNNNATCTACACTTA | 5'-Phosphorylation |
| oS4-F | **TAGA**TNNNNNNNNNNNNNNNNNNNNNNNTAATTTCTACT | 5'-Phosphorylation |
| oS4-R | **ACTT**AGTAGAAATTANNNNNNNNNNNNNNNNNNNNNNNA | 5'-Phosphorylation |
| oS5-F | **AAGT**GTAGATNNNNNNNNNNNNNNNNNNNNNNNTAATTTCTACTA | 5'-Phosphorylation |
| oS5R | **CACT**TAGTAGAAATTANNNNNNNNNNNNNNNNNNNNNNNATCTAC | 5'-Phosphorylation |
| oS6-F | **AGTG**TAGATNNNNNNNNNNNNNNNNNNNNNNNG | 5'-Phosphorylation |
| oS6-R | **CGGC**CNNNNNNNNNNNNNNNNNNNNNNNATCTA | 5'-Phosphorylation |

**Generation of short inserts by annealing two 5'-phosphorylated oligos (primers)**

oS1-F + R = Ins-S1
oS2-F + R = Ins-S2
oS3-F + R = Ins-S3
oS4-F + R = Ins-S4
oS5-F + R = Ins-S5
oS6-F + R = Ins-S6

**BbsI-based Golden Gate Cloning**

pAGC-Lb12-P1Bb + Ins-S1 + Ins-S2 + Ins-S3 + Ins-S4 + Ins-S5 + Ins-S6 = **pAGC-S1–S6**

**Colony PCR primers:** oS1-F + oS6-R = 662-bp
**Sequencing primers:** M13F and M13R

## Final cassette for expression of 6×crRNAs for LbCas12a

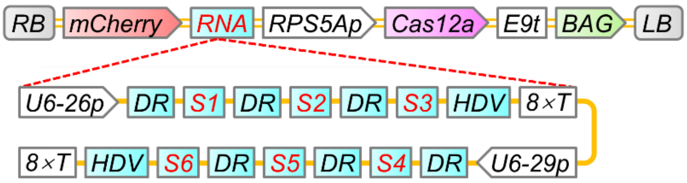


U6-26p-DR-S1-DR-S2-DR-S3-HDV-U6-29t-U6-29p-DR-S4-DR-S5-DR-S6-HDV-U6-26t
CGACTTGCCTTCCGCACAATACATCATTTCTTCTTAGCTTTTTTTCTTCTTCTTCGTTCATACAGTTTTT
TTTTGTTTATCAGCTTACATTTTCTTGAACCGTAGCTTTCGTTTTCTTCTTTTTAACTTTCCATTCGGAG
TTTTTGTATCTTGTTTCATAGTTTGTCCCAGGATTAGAATGATTAGGCATCGAACCTTCAAGAATTTGAT
TGAATAAAACATCTTCATTCTTAAGATATGAAGATAATCTTCAAAAGGCCCCTGGGAATCTGAAAGAAG
AGAAGCAGGCCCATTTATATGGGAAAGAACAATAGTATTTCTTATATAGGCCCATTTAAGTTGAAAACA
ATCTTCAAAAGTCCCACATCGCTTAGATAAGAAAACGAAGCTGAGTTTATATACAGCTAGAGTCGAAG
TAGTGATTGTAATTTCTACTAAGTGTAGATNNNNNNNNNNNNNNNNNNNNNNNTAATTTCTACTAAGTG
TAGATNNNNNNNNNNNNNNNNNNNNNNNTAATTTCTACTAAGTGTAGATNNNNNNNNNNNNNNNNNN
NNNNNTTGGCCGGCATGGTCCCAGCCTCCTCGCTGGCGCCGGCTGGGCAACATGCTTCGGCATGG
CGAATGGGACTTTTTTTTGGATAGAATTTCCCAGCTTTTTTGCGTGTTTCAGCTCTCATGATCCTTAAT
CCAAACTACTGCAGCCTGACAGACAAATGAGGATGCAAACAATTTTAAAGTTTATCTAACGCTAGCTG
TTTTGTTTCTTCTCTCTGGTGCACCAACGACGGCGTTTTCTCAATCATAAAGAGGCTTGTTTTACTTAA
GGCCAATAATGTTGATGGATCGAAAGAAGAGGGCTTTTAATAAACGAGCCCGTTTAAGCTGTAAACGA
TGTCAAAAACATCCCACATCGTTCAGTTGAAAATAGAAGCTCTGTTTATATATTGGTAGAGTCGACTAA
GAGATTGTAATTTCTACTAAGTGTAGATNNNNNNNNNNNNNNNNNNNNNNNTAATTTCTACTAAGTGT
AGATNNNNNNNNNNNNNNNNNNNNNNNTAATTTCTACTAAGTGTAGATNNNNNNNNNNNNNNNNNN
NNNNNGGCCGGCATGGTCCCAGCCTCCTCGCTGGCGCCGGCTGGGCAACATGCTTCGGCATGGC
GAATGGGACTTTTTTTTGCAAAATTTTCCAGATCGATTTCTTCTTCCTCTGTTCTTCGGCGTTCAATTT
CTGGGGTTTTCTCTTCGTTTTCTGTAACTGAAACCTAAAATTTGACCTAAAAAAAATCTCAAATAATATG
ATTCAGTGGTTTTGTACTTTTCAGTTAGTTGAGTTTTGCAGTTCCGATGAGATAAACCAATA

(Two extra T bases in S3 do not affect editing efficiency)

**BsaI-based Golden Gate Cloning**

pBG-ttLbUV2 + pAGC-S1–S6 = **pBG-S1–S6**

**Colony PCR and sequencing primers**

| | |
|---|---|
| U6-26p-F | TGTCCCAGGATTAGAATGATTAGGC |
| U6-26t-R | CCCCAGAAATTGAACGCCGAAGAAC |
| U6-26p-F + U6-26t-R = 1.1-kb | |

## Cloning cassette for assembly of Cas12i3 crRNA

U6-26p-i3DR-BsaI-BsaI-HDV-U6-26t

CGACTTGCCTTCCGCACAATACATCATTTCTTCTTAGCTTTTTTTCTTCTTCTTCGTTCATACAGTTTTT
TTTTGTTTATCAGCTTACATTTTCTTGAACCGTAGCTTTCGTTTTCTTCTTTTTAACTTTCCATTCGGAG
TTTTTGTATCTTGTTTCATAGTTTGTCCCAGGATTAGAATGATTAGGCATCGAACCTTCAAGAATTTGAT
TGAATAAAACATCTTCATTCTTAAGATATGAAGATAATCTTCAAAAGGCCCCTGGGAATCTGAAAGAAG
AGAAGCAGGCCCATTTATATGGGAAAGAACAATAGTATTTCTTATATAGGCCCATTTAAGTTGAAAACA
ATCTTCAAAAGTCCCACATCGCTTAGATAAGAAAACGAAGCTGAGTTTATATACAGCTAGAGTCGAAG
TAGTGATTGAGAGAATGTGTGCATAGTCACACAGAGACCNNNNNNNNNNNNNNNNNNNNNNNGGTC
TCAGCCGGCATGGTCCCAGCCTCCTCGCTGGCGCCGGCTGGGCAACATGCTTCGGCATGGCGAAT
GGGACTTTTTTTTGCAAAATTTTCCAGATCGATTTCTTCTTCCTCTGTTCTTCGGCGTTCAATTTCTGG
GGTTTTCTCTTCGTTTTCTGTAACTGAAACCTAAAATTTGACCTAAAAAAAATCTCAAATAATATGATTC
AGTGGTTTTGTACTTTTCAGTTAGTTGAGTTTTGCAGTTCCGATGAGATAAACCAATA

## Final cassette for expression of a Cas12i3 crRNA

U6-26p-i3DR-Target-HDV-U6-26t

CGACTTGCCTTCCGCACAATACATCATTTCTTCTTAGCTTTTTTTCTTCTTCTTCGTTCATACAGTTTTT
TTTTGTTTATCAGCTTACATTTTCTTGAACCGTAGCTTTCGTTTTCTTCTTTTTAACTTTCCATTCGGAG
TTTTTGTATCTTGTTTCATAGTTTGTCCCAGGATTAGAATGATTAGGCATCGAACCTTCAAGAATTTGAT
TGAATAAAACATCTTCATTCTTAAGATATGAAGATAATCTTCAAAAGGCCCCTGGGAATCTGAAAGAAG
AGAAGCAGGCCCATTTATATGGGAAAGAACAATAGTATTTCTTATATAGGCCCATTTAAGTTGAAAACA
ATCTTCAAAAGTCCCACATCGCTTAGATAAGAAAACGAAGCTGAGTTTATATACAGCTAGAGTCGAAG
TAGTGATTGAGAGAATGTGTGCATAGTCACACNNNNNNNNNNNNNNNNNNNNNNNGGCCGGCATGG
TCCCAGCCTCCTCGCTGGCGCCGGCTGGGCAACATGCTTCGGCATGGCGAATGGGACTTTTTTTTG
CAAAATTTTCCAGATCGATTTCTTCTTCCTCTGTTCTTCGGCGTTCAATTTCTGGGGTTTTCTCTTCGT
TTTCTGTAACTGAAACCTAAAATTTGACCTAAAAAAAATCTCAAATAATATGATTCAGTGGTTTTGTACT
TTTCAGTTAGTTGAGTTTTGCAGTTCCGATGAGATAAACCAATA

**Primers**

| | | |
|---|---|---|
| oCas12i3T-F | **ACAC**NNNNNNNNNNNNNNNNNNNNNNNG | 5'-Phosphorylation |
| oCas12i3T-R | **CGGC**CNNNNNNNNNNNNNNNNNNNNNNN | 5'-Phosphorylation |

**Generation of short inserts by annealing two 5'-phosphorylated oligos (primers)**

oCas12i3T-F+R = Ins1-i3S1

**BsaI-based Golden Gate Cloning**

pBG-Cas12i3V1-Bs + Ins1-i3S1= **pi3m5V1-S1**

pBG-Cas12i3V2-Bs + Ins1-i3S1= **pi3m5V2-S1**

## Cloning cassette for assembly of AsCas12f sgRNA

U6-26p-sgRNA scaffold-BsaI-BsaI-HDV-U6-26t

CGACTTGCCTTCCGCACAATACATCATTTCTTCTTAGCTTTTTTTCTTCTTCTTCGTTCATACAGTTTTT
TTTTGTTTATCAGCTTACATTTTCTTGAACCGTAGCTTTCGTTTTCTTCTTTTTAACTTTCCATTCGGAG
TTTTTGTATCTTGTTTCATAGTTTGTCCCAGGATTAGAATGATTAGGCATCGAACCTTCAAGAATTTGAT
TGAATAAAACATCTTCATTCTTAAGATATGAAGATAATCTTCAAAAGGCCCCTGGGAATCTGAAAGAAG
AGAAGCAGGCCCATTTATATGGGAAAGAACAATAGTATTTCTTATATAGGCCCATTTAAGTTGAAAACA
ATCTTCAAAAGTCCCACATCGCTTAGATAAGAAAACGAAGCTGAGTTTATATACAGCTAGAGTCGAAG
TAGTGATTGATTCGTCGGTTCAGCGACGATAAGCCGAGAAGTGCCAATAAAACTGTTAAGTGGTTTG
GTAACGCTCGGTAAGGTCCGAAAGGAGAACCACTGAACTGAGACCNNNNNNNNNNNNNNNNNNNNN
GGTCTCAGCCGGCATGGTCCCAGCCTCCTCGCTGGCGCCGGCTGGGCAACATGCTTCGGCATGGC
GAATGGGACTTTTTTTTGCAAAATTTTCCAGATCGATTTCTTCTTCCTCTGTTCTTCGGCGTTCAATTT
CTGGGGTTTTCTCTTCGTTTTCTGTAACTGAAACCTAAAATTTGACCTAAAAAAAATCTCAAATAATATG
ATTCAGTGGTTTTGTACTTTTCAGTTAGTTGAGTTTTGCAGTTCCGATGAGATAAACCAATA

## Final cassette for expression of an AsCas12f sgRNA

U6-26p- sgRNA scaffold-Target-HDV-U6-26t

CGACTTGCCTTCCGCACAATACATCATTTCTTCTTAGCTTTTTTTCTTCTTCTTCGTTCATACAGTTTTT
TTTTGTTTATCAGCTTACATTTTCTTGAACCGTAGCTTTCGTTTTCTTCTTTTTAACTTTCCATTCGGAG
TTTTTGTATCTTGTTTCATAGTTTGTCCCAGGATTAGAATGATTAGGCATCGAACCTTCAAGAATTTGAT
TGAATAAAACATCTTCATTCTTAAGATATGAAGATAATCTTCAAAAGGCCCCTGGGAATCTGAAAGAAG
AGAAGCAGGCCCATTTATATGGGAAAGAACAATAGTATTTCTTATATAGGCCCATTTAAGTTGAAAACA
ATCTTCAAAAGTCCCACATCGCTTAGATAAGAAAACGAAGCTGAGTTTATATACAGCTAGAGTCGAAG
TAGTGATTGATTCGTCGGTTCAGCGACGATAAGCCGAGAAGTGCCAATAAAACTGTTAAGTGGTTTG
GTAACGCTCGGTAAGGTCCGAAAGGAGAACCACTGAACNNNNNNNNNNNNNNNNNNNNNCGCCGGC
ATGGTCCCAGCCTCCTCGCTGGCGCCGGCTGGGCAACATGCTTCGGCATGGCGAATGGGACTTTTT
TTTGCAAAATTTTCCAGATCGATTTCTTCTTCCTCTGTTCTTCGGCGTTCAATTTCTGGGGTTTTCTCT
TCGTTTTCTGTAACTGAAACCTAAAATTTGACCTAAAAAAAATCTCAAATAATATGATTCAGTGGTTTTG
TACTTTTCAGTTAGTTGAGTTTTGCAGTTCCGATGAGATAAACCAATA

**Primers**

| | | |
|---|---|---|
| oCas12fT-F | **GAAC**NNNNNNNNNNNNNNNNNNNNNC | 5'-Phosphorylation |
| oCas12fT-R | **CGGC**GNNNNNNNNNNNNNNNNNNNNN | 5'-Phosphorylation |

**Generation of short inserts by annealing two 5'-phosphorylated oligos (primers)**

oCas12fT-F+R = Ins1-12fS1

**BsaI-based Golden Gate Cloning**

pBG-YHAM-Bs + Ins1-12fS1= **pYHAM-S1**

pBG-HKRA-Bs + Ins1-12fS1= **pHKRA-S1**
